# Comparison of the efficacy and safety of third-line treatments for metastatic colorectal cancer: a systematic review and network meta-analysis

**DOI:** 10.3389/fonc.2023.1269203

**Published:** 2023-09-21

**Authors:** Loulu Gao, Lin Tang, Zixuan Hu, Jieqiong Peng, Xiaoqian Li, Bo Liu

**Affiliations:** ^1^ School of Clinical Medicine, Weifang Medical University, Weifang, China; ^2^ Department of Oncology, Shandong Cancer Hospital and Institute, Shandong First Medical University and Shandong Academy of Medical Sciences, Jinan, China; ^3^ Department of Oncology, Shandong First Medical University and Shandong Academy of Medical Sciences, Jinan, China

**Keywords:** colorectal cancer, third-line, neoplasm metastasis, network meta-analysis (NMA), treatment

## Abstract

**Background:**

The objective of this study is to evaluate the efficacy and safety of different third-line treatment regimens for metastatic colorectal cancer (mCRC) through a comprehensive analysis and network meta-analysis (NMA). Additionally, the study aims to provide guidance on selecting appropriate third-line systemic treatment regimens for patients with mCRC.

**Methods:**

We conducted a search of the PubMed, Embase, Web of Science, and Cochrane Central Register of Controlled Trials databases from January 1, 2005, to May 20, 2023, to include phase II/III randomized clinical trials (RCTs) of third-line treatments for mCRC. The primary outcome assessed in the NMA was median overall survival (mOS), and other outcomes included median progression-free survival (mPFS), disease control rate (DCR), and grade 3 or higher adverse events (≥3AEs).

**Results:**

Ultimately, nine phase II/III RCTs involving five treatment regimens were included in this study. Trifluridine/tipiracil (TAS-102) plus bevacizumab (hazard ratio [HR] 0.41, 95% credible interval [CrI] 0.32-0.52) was found to be the most effective treatment for mOS compared to best supportive care (BSC). TAS-102 plus bevacizumab also significantly improved mPFS compared to BSC (HR 0.20, 95% CrI 0.16-0.25). In terms of adverse events (AEs), TAS-102 (RR 0.52, 95% CrI 0.35-0.74) had a lower incidence of ≥3AEs compared to fruquintinib, but fruquintinib (RR 1.79, 95% CrI 1.10-3.11) showed better improvement in DCR than TAS-102. Subgroup analysis using the Bayesian surface under the cumulative ranking curve (SUCRA) ranked the regimens based on the OS benefit. The results indicated that TAS-102 plus bevacizumab ranked first across age, gender, Eastern Cooperative Oncology Group performance status (ECOG PS), and time from initial diagnosis of metastatic disease to randomization.

**Conclusion:**

TAS-102, fruquintinib, TAS-102 plus bevacizumab, the regorafenib standard dose regimen (regorafenib), and the regorafenib dose-escalation regimen (regorafenib 80+) all demonstrated improved OS and PFS compared to BSC in mCRC patients. However, TAS-102 plus bevacizumab may be the optimal choice for third-line treatment in mCRC patients.

**Systematic review registration:**

https://www.crd.york.ac.uk/prospero/display_record.php, CRD42023434929.

## Introduction

Colorectal cancer (CRC) is the third most common cancer in men and the second most common in women (1). It accounts for approximately 10% of all cancer diagnoses and cancer-related deaths worldwide (2). Early-stage CRC patients often lack typical symptoms, and 20%–30% of them already have metastatic disease at the time of diagnosis (3). The prognosis for metastatic CRC (mCRC) is poor, with a 5-year survival rate of less than 20% (4).

The main treatments for early-stage CRC patients are surgery, radiotherapy, and chemotherapy. For patients with mCRC, first- and second-line treatments typically involve oxaliplatin or irinotecan combined with a fluoropyrimidine (5-fluorouracil or capecitabine), often in combination with targeted drug therapy such as vascular endothelial growth factor (VEGF) inhibitors or epidermal growth factor receptor (EGFR) inhibitors for patients with RAS wild-type (5, 6). However, most patients with mCRC eventually become insensitive or non-responsive to these treatments or intolerant to multiple cycles, leading to the need for third-line therapy. Therefore, the choice of appropriate treatment options plays a crucial role in prolonging survival.

Currently, several drugs have been approved for the standard third-line treatment of mCRC through validation in clinical trials. Regorafenib is the first small-molecule kinase inhibitor approved for the third-line treatment of mCRC. It improves patient survival by inhibiting multiple tumor growth-promoting protein kinases involved in tumor cell production, tumor angiogenesis, and maintenance of tumor microenvironment (TME) signaling (7). Trifluridine/tipiracil (TAS-102) is an oral cytotoxic antitumor drug composed of trifluridine (FTD) and tipiracil hydrochloride (TPI) in a specific ratio (8). It acts by incorporating into tumor cell DNA, thereby inhibiting tumor cell growth and proliferation (9). The presence of a thymidine phosphorylase inhibitor protects FTD from degradation and increases the concentration of the antitumor drug component (10). In the RECOURSE study, the TAS-102 group exhibited significantly higher disease control rates (DCR) (44% *vs*. 16%), longer survival (7.1 months *vs*. 5.3 months), and a 32% reduction in the risk of patient death compared to the best supportive care (BSC) group (11). The efficacy of TAS-102 was further confirmed in the 2013 TERRA study involving Asian populations (12). Fruquintinib, a highly selective oral tyrosine kinase inhibitor (TKI), gained global approval for the first time in China in 2018 for the treatment of mCRC patients who have failed at least second-line therapy, leading to benefits in both overall survival (OS) and progression-free survival (PFS) (13).

While most randomized controlled trials (RCTs) have assessed the efficacy and safety of these treatments compared to the BSC group, there is a lack of head-to-head comparisons between different treatment regimens. As a result, the selection of appropriate third-line treatment regimens for mCRC patients remains an unresolved issue. The objective of this study is to analyze the treatment effects, adverse events (AEs), and impact on relevant subgroups of various regimens through a systematic review and network meta-analysis (NMA) in the absence of direct comparisons. The aim is to evaluate the efficacy and tolerability of each regimen. The results of this study can help provide some clinical reference for the selection of third-line treatment options for mCRC patients.

## Materials and methods

This study adheres to the Preferred Reporting Items for Systematic Reviews and Meta-analyses (PRISMA) statement extension for network meta-analysis (NMA) (**Supplementary Table 1**) (14).

### Literature search strategies and eligibility criteria

A comprehensive search was performed in the PubMed, Embase, Web of Science, and Cochrane Central Register of Controlled Trials databases from January 1, 2005, to May 20, 2023, using the search strategy outlined in **Supplementary Table 2**. We included phase II/III randomized controlled trials (RCTs) focusing on third-line treatment for metastatic colorectal cancer (mCRC) in the network meta-analysis (NMA). The inclusion criteria for this study were as follows: 1) phase II/III RCTs; 2) histologically confirmed mCRC in patients included in the trial; and 3) The hazard ratios (HRs) and 95% credible intervals (CrIs) for overall survival (OS) and progression-free survival (PFS), disease control rate (DCR), and adverse events (AEs) were available. Exclusion criteria: 1) non-RCTs, single-arm design studies, and dose-finding studies; 2) trial results limited to specific patient groups only, e.g., the patient group was elderly only, male only, or female only;3) studies with insufficient published data for analysis or unpublished final results.

### Data extraction and risk of bias assessment

The following information was extracted from the articles: study title, study ID, publication year, first author, number of study subjects, baseline characteristics, OS, PFS, DCR, and grade 3 or higher adverse events (≥3AEs). The risk of bias in the included trials was assessed using the Cochrane risk of bias tool, which assessed seven aspects: random sequence generation, allocation concealment, blinding of participants and personnel, blinding of outcome assessment, incomplete outcome data, selective reporting, and other sources of bias. Two reviewers (LLG and ZXH) independently conducted data extraction and assessed the risk of bias in the included studies. Any disagreements were resolved by a third reviewer (BL).

### Statistical analysis

The primary outcome of this study was mOS. Secondary outcomes were median progression-free survival (mPFS), DCR, and ≥3AEs. The statistical heterogeneity between treatment effects across RCTs was assessed using the I^2^ statistic. I^2^ values below 25%, between 25% and 50%, or above 50% indicated low, moderate, and high heterogeneity, respectively (15). A network plot was generated using Stata 16.0 to visually display the comparative relationships among the various treatment regimens. Fixed and random effect models were considered and compared using deviance information criteria (DIC). If the difference in DIC between the random model and the fixed model was less than 5, the fixed model should be selected (16). The NMA was performed within a Bayesian framework using the Markov chain Monte Carlo simulation technique implemented with the GEMTC package in R-Statistics and the J.A.G.S. program (17). Each analysis involved 20,000 sample iterations with 5,000 burn-in cycles and a thinning interval of 1. Model convergence was assessed using Brooks-Gelman-Rubin diagnostic plots and trace plots (18). To estimate the probability of each treatment ranking, we calculated the surface under the cumulative ranking curve (SUCRA). A higher SUCRA value indicates a greater likelihood of a treatment regimen being the preferred option (19).

## Results

### Literature search and study characteristics

**Figure 1:**
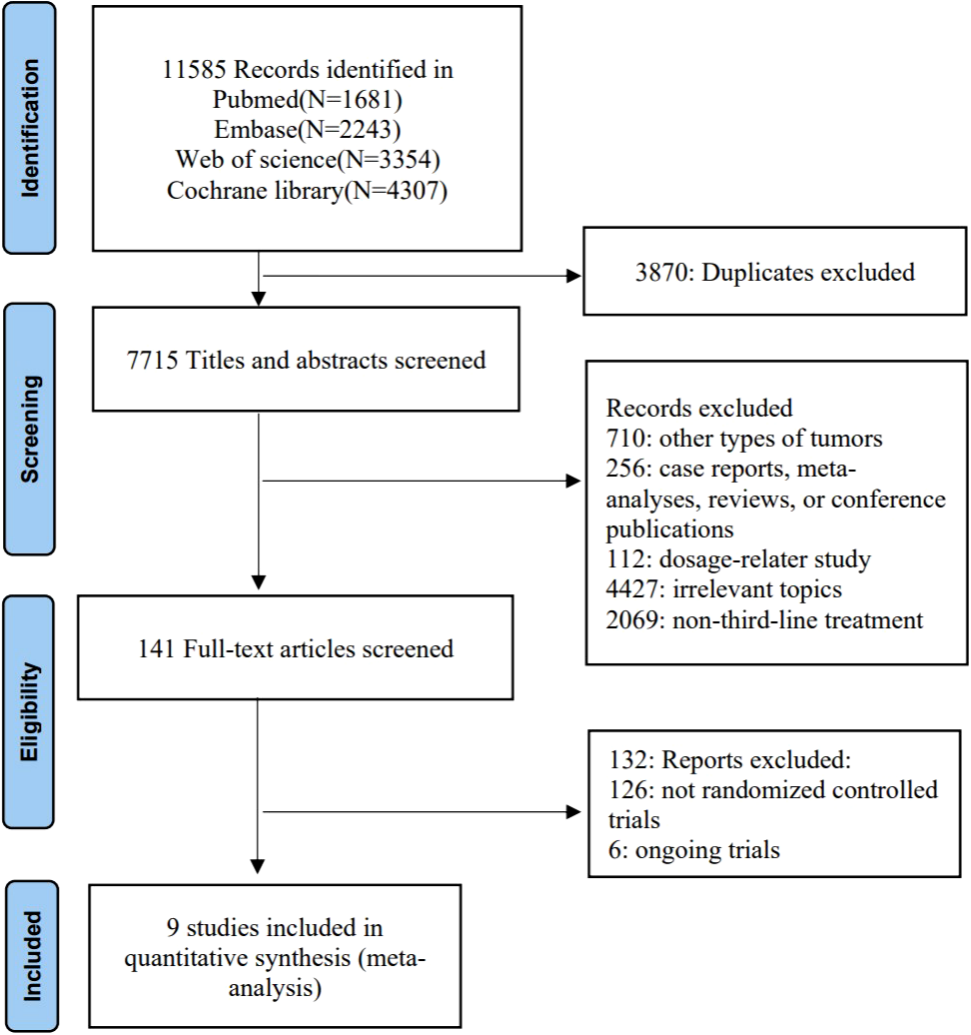
Screening and selection process.

**Figure 2:**
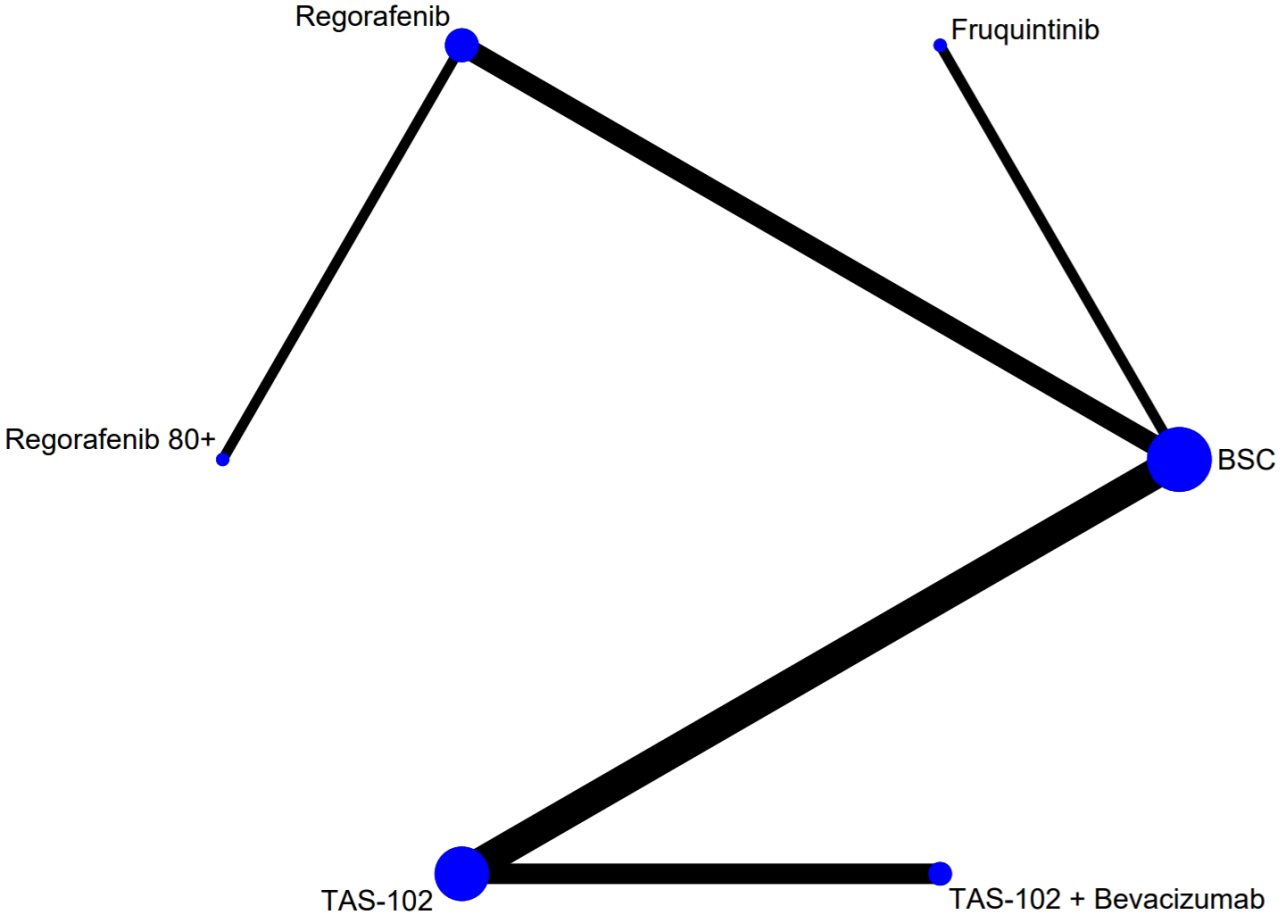
The network analysis diagram. Each circular node represented a treatment measure. The size of the nodes indicated the number of people involved in that treatment. The line between the two nodes represented the existence of a direct comparison between the two treatment options, and the thickness of the line indicated the number of direct comparisons. TAS-102, Trifluridine/Tipiracil; BSC, best supportive care.

The flow chart depicting the study selection process is shown in **Figure 1**. Ultimately, we included nine phase II/III randomized controlled trials (RCTs) (11–13, 20–25), involving a total of 3456 patients and encompassing five treatment regimens. These treatments included chemotherapy (TAS-102), chemotherapy in combination with an anti-angiogenic agent (TAS-102 plus bevacizumab), best supportive care (BSC), and anti-angiogenic agents (regorafenib, regorafenib 80+, and fruquintinib). The included studies of regorafenib included two different dosage regimens: one of 160 mg/day, administered orally for 21 consecutive days over a 28-day treatment cycle (regorafenib); the other used a treatment regimen with a starting dose of 80 mg/day, which was increased by 40 mg per week up to 160 mg/day in the absence of any significant drug-related adverse effects (regorafenib 80+). The network diagram for direct and indirect comparison of all treatments is shown in **Figure 2**. The baseline characteristics of the study are shown in **Table 1**. Our NMA satisfied the transitivity assumption that the population baseline is relatively stable among the different interventions included in the study. (**Supplementary Figure 7**).

**Table 1:** Baseline characteristics of studies included in the systematic review with Bayesian network meta-analysis of third-line treatments for metastatic colorectal cancer.

Study(phase)	Trial name	Register	Study design	Sample size	Median age	Previous chemotherapy	Intervention arm	Control arm	Primary endpoint
Grothey A et al. (III)	CORRECT	NCT01103323	Randomized double-blind trial	505/255	61/61	One or more	Regorafenib at 160 mg/day	Placebo+BSC	OS
Li J et al. (III)	CONCUR	NCT01584830	Randomized double-blind trial	136/68	57·5/55·5	Two or more	Regorafenib at 160 mg/day	Placebo+BSC	OS
Mayer RJ et al. (III)	RECOURSE	NCT01607957	Randomized double-blind trial	534/266	63/63	Two or more	TAS-102 at 35 mg/m^2^ twice daily	Placebo+BSC	OS
Xu J et al. (III)	TERRA	NCT01955837	Randomized double-blind trial	271/135	58/56	Two or more	TAS-102 at 35 mg/m^2^ twice daily	Placebo+BSC	OS
Li J et al. (III)	FRESCO	NCT02314819	Randomized double-blind trial	278/138	55.0/57.0	Two or more	Fruquintinib at 5mg/day	Placebo+BSC	OS
Yoshino T et al. (II)	NA	JapicCTI-090880	Randomized double-blind trial	112/57	63/62	Two or more	TAS-102 at 35 mg/m^2^ twice daily	Placebo+BSC	OS
Bekaii-Saab TS et al. (II)	ReDOS	NCT02368886	Randomized open-label trial	54/62	62/61	Two or more	The starting dose of regorafenib was 80 mg/day in week 1, 120 mg/day in week 2, and 160 mg/day in week 3 for cycle 1. Weekly incremental dose escalation occurred up to the maximum of 160 mg/day if no significant drug-related toxicities were observed	Regorafenib at 160 mg/day	The proportion of patients in each group who completed two cycles of treatment and initiated the third cycle
Pfeiffer P et al. (II)	NA	EudraCT 2016–005241–23	Randomized open-label trial	46/47	64/67	One or more	TAS-102 at 35 mg/m^2^ twice daily plus bevacizumab at 5 mg/kg intravenously on days 1 and 15 every 28 days	TAS-102 at 35 mg/m2 twice daily	PFS
Prager GW et al. (III)	SUNLIGHT	NCT04737187	Randomized open-label trial	246/246	62/64	One or more	TAS-102 at 35 mg/m^2^ twice daily plus bevacizumab at 5 mg/kg intravenously on days 1 and 15 every 28 days	TAS-102 at 35 mg/m2 twice daily	OS

NA, not available; TAS-102, Trifluridine/Tipiracil; BSC, best supportive care; OS, overall survival; PFS, progression-free survival.

### Overall outcomes

**Figure 3:**
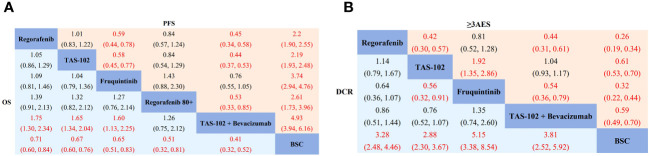
Network meta-analysis of the third-line treatments for mCRC. **(A)** Pooled hazard ratio (HR) [95% CrIs (credible intervals)] for overall survival (OS) and progression-free survival (PFS) in the overall population. **(B)** Pooled relative risk (RR) (95% CrIs) for disease control rate (DCR) and grade 3 or higher adverse events (≥3AEs) in the overall population. TAS-102, Trifluridine/Tipiracil; BSC, best supportive care.

Regarding overall survival (OS), compared to BSC, regorafenib (HR 0.71, 95% CrI 0.60-0.84), TAS-102 (HR 0.67, 95% CrI 0.60-0.76), fruquintinib (HR 0.65, 95% CrI 0.51-0.83), regorafenib 80+ (HR 0.51, 95% CrI 0.32-0.81), and TAS-102 plus bevacizumab (HR 0.41, 95% CrI 0.32-0.52) demonstrated superior efficacy (**Figure 3A**). According to the SUCRA results, TAS-102 plus bevacizumab (0.96) had the highest probabilities of ranking first, followed by regorafenib 80+ (0.76), fruquintinib (0.50), TAS-102 (0.44), and regorafenib (0.33) (**Supplementary Figure 1A**). In terms of progression-free survival (PFS), compared to BSC, regorafenib (HR 0.45, 95% CrI 0.39-0.53), TAS-102 (HR 0.46, 95% CrI 0.40-0.52), fruquintinib (HR 0.27, 95% CrI 0.21-0.34), regorafenib 80+ (HR 0.38, 95% CrI 0.25-0.58), and TAS-102 plus bevacizumab (HR 0.21, 95% CrI 0.16-0.25) were all more effective than BSC. TAS-102 plus bevacizumab also showed better PFS than regorafenib 80+ (HR 0.53, 95% CrI 0.33-0.85) (**Figure 3A**). The SUCRA value for TAS-102 plus bevacizumab (0.99) was higher than the other treatment regimens, followed by fruquintinib (0.80), regorafenib 80+ (0.53), and TAS-102 (0.34) (**Supplementary Figure 1A**). In terms of disease control rate (DCR) compared to BSC, regorafenib (RR: 3.28, 95% CrI 2.48-4.46), TAS-102 (RR: 2.88, 95% CrI 2.30-3.67), fruquintinib (RR: 5.15, 95% CrI 3.38-8.54), and TAS-102 plus bevacizumab (RR: 3.81, 95% CrI 2.52-5.92) demonstrated superiority. Fruquintinib (RR: 1.79, 95% CrI 1.10-3.11) was superior to TAS-102 (**Figure 3B**). The SUCRA values, in descending order, were as follows: fruquintinib (0.94), TAS-102 plus bevacizumab (0.71), regorafenib (0.52), and TAS-102 (0.33) (**Supplementary Figure 1A**). Regarding adverse events (AEs) with grade ≥3, the incidence rates of regorafenib (RR: 3.88, 95% CrI 2.98-5.23), TAS-102 (RR: 1.63, 95% CrI 1.43-1.88), fruquintinib (RR: 3.13, 95% CrI 2.26-4.59), and TAS-102 plus bevacizumab (RR: 1.72, 95% CrI 1.42-2.04) were all higher than BSC (**Figure 3B**). Gastrointestinal and hematologic toxicities were the major AEs associated with TAS-102 plus bevacizumab, although their incidence rates in the network meta-analysis were relatively low (**Supplementary Figure 3**).

### NMA of age, gender, ECOG and region subgroup

In the age subgroup, for patients aged ≥65 years, TAS-102 (HR 0.57, 95% CrI 0.46-0.70) and TAS-102 plus bevacizumab (HR 0.33, 95% CrI 0.23-0.49) significantly prolonged survival compared to BSC. TAS-102 plus bevacizumab was also superior to regorafenib (HR 2.44, 95% CrI 1.48-4.02), TAS-102 (HR 1.71, 95% CrI 1.23-2.38), and fruquintinib (HR 2.85, 95% CrI 1.45-5.54). For patients aged <65 years, regorafenib (HR 0.67, 95% CrI 0.55-0.82), TAS-102 (HR 0.79, 95% CrI 0.67-0.93), fruquintinib (HR 0.56, 95% CrI 0.43-0.73), and TAS-102 plus bevacizumab (HR 0.51, 95% CrI 0.36-0.71) all significantly improved OS compared to BSC. Fruquintinib (HR 0.71, 95% CrI 0.52-0.97) and TAS-102 plus bevacizumab (HR 0.65, 95% CrI 0.48-0.87) were also superior to TAS-102 (**Supplementary Figure 4A**). In the gender subgroup, regorafenib (HR 0.74, 95% CrI 0.59-0.93), TAS-102 (HR 0.70, 95% CrI 0.59-0.82), fruquintinib (HR 0.52, 95% CrI 0.39-0.70), and TAS-102 plus bevacizumab (HR 0.42, 95% CrI 0.30-0.58) demonstrated an OS benefit in male patients compared to BSC. In female patients, regorafenib (HR 0.66, 95% CrI 0.51-0.86), TAS-102 (HR 0.72, 95% CrI 0.58-0.88), and TAS-102 plus bevacizumab (HR 0.42, 95% CrI 0.29-0.59) showed longer OS compared to BSC, except for fruquintinib (**Supplementary Figure 4B**). In patients with ECOG PS=0, regorafenib (HR 0.69, 95% CrI 0.53-0.90), TAS-102 (HR 0.71, 95% CrI 0.59-0.87), fruquintinib (HR 0.49, 95% CrI 0.31-0.79), and TAS-102 plus bevacizumab (HR 0.47, 95% CrI 0.33-0.68) prolonged survival significantly compared to BSC. In patients with ECOG PS=1, regorafenib (HR 0.69, 95% CrI 0.56-0.86), TAS-102 (HR 0.69, 95% CrI 0.58-0.83), fruquintinib (HR 0.68, 95% CrI 0.52-0.90), and TAS-102 plus bevacizumab (HR 0.39, 95% CrI 0.21-0.72) all demonstrated significantly better OS than BSC (**Supplementary Figure 4C**).

### NMA of different KRAS status subgroup

In the KRAS wild-type subgroup, regorafenib (HR 0.64, 95% CrI 0.49-0.84), TAS-102 (HR 0.65, 95% CrI 0.55-0.78), and fruquintinib (HR 0.56, 95% CrI 0.40-0.78) demonstrated superior efficacy compared to BSC. In KRAS mutant patients, TAS-102 (HR 0.76, 95% CrI 0.63-0.92) achieved a significant OS benefit compared to BSC, while regorafenib and fruquintinib did not differ significantly from BSC (**Supplementary Figures 1A**, **4D**).

### NMA of primary sites subgroup

In patients with a primary tumor site in the colon, regorafenib (HR 0.71, 95% CrI 0.56-0.89) and TAS-102 (HR 0.70, 95% CrI 0.59-0.87) showed a benefit in OS compared to BSC. However, fruquintinib did not improve OS, and TAS-102 had a higher SUCRA value compared to regorafenib. In patients with rectal cancer, TAS-102 (HR 0.65, 95% CrI 0.53-0.81) and fruquintinib (HR 0.59, 95% CrI 0.41-0.86) were superior to BSC. Regarding SUCRA values, fruquintinib (0.87) was higher than TAS-102 (0.76) and regorafenib (0.23) (**Supplementary Figures 1A**, **4E**).

### NMA of time since diagnosis of the first metastases

In the subgroup with a time of less than 18 months, regorafenib (HR 0.68, 95% CrI 0.49-0.93) and the combination of TAS-102 plus bevacizumab (HR 0.44, 95% CrI 0.29-0.67) demonstrated benefits in terms of OS compared to BSC. The combination of TAS-102 with bevacizumab was superior to TAS-102 alone (HR 1.94, 95% CrI 1.39-2.70). In the subgroup with a time greater than or equal to 18 months, regorafenib (HR 0.73, 95% CrI 0.60-0.88), TAS-102 (HR 0.65, 95% CrI 0.55-0.77), and TAS-102 plus bevacizumab (HR 0.46, 95% CrI 0.33-0.64) all improved OS compared to BSC. Furthermore, the combination of TAS-102 plus bevacizumab was superior to regorafenib (HR 1.58, 95% CrI 1.08-2.32) and TAS-102 alone (HR 1.42, 95% CrI 1.06-1.89) (**Supplementary Figure 4F**).

### Rank probabilities

According to the SUCRA values, the ranking of different treatment options in different subgroups and the Bayesian ranking curve were estimated (**Supplementary Figure 1B**). The Bayesian ranking results were consistent with the NMA. TAS-102 plus bevacizumab had the highest SUCRA value for OS and PFS, indicating that it is a relatively effective treatment option for improving OS and PFS. Among regorafenib, TAS-102, fruquintinib, and TAS-102 plus bevacizumab, fruquintinib ranked first in DCR, and regorafenib ranked first in terms of ≥3AEs, indicating relatively higher toxicity. In the subgroups of age, gender, ECOG PS, and time since diagnosis of the first metastases, TAS-102 plus bevacizumab ranked first. In the subgroup of primary tumor site, compared with regorafenib and fruquintinib, TAS-102 ranked first in the colon group, while fruquintinib ranked first in the rectal group. Some treatment options were missing from subgroup analyses, resulting in relatively incomplete rankings.

### Risk of bias assessment, model convergence, heterogeneity and inconsistency analysis

According to the results of the risk of bias assessment, the majority of RCTs had a low risk of bias. Please refer to **Supplementary Figure 5** for the bias risk assessment chart. As seen from the trajectory plots and Brooks-Gelman-Rubin diagnostic plots, the chosen model demonstrated acceptable convergence (**Supplementary Figure 6**). The statistical heterogeneity of the studies, both in the primary and secondary outcomes, ranged from low to moderate (I^2^ < 50%, ranging from 1% to 50%) (**Supplementary Table 3**). In most comparisons, the fit of the consistency model was similar to or better than the inconsistency model (**Supplementary Table 3**).

## Discussion

Regorafenib and TAS-102 have emerged as standard third-line treatments for refractory mCRC. The approval of fruquintinib in China in 2018, based on the FRESCO study, has provided an additional treatment option (13). Previous meta-analyses have reported comparable efficacy between regorafenib and TAS-102, with regorafenib showing relatively higher toxicity, which is consistent with the findings of this study (26, 27). A NMA presented at ESMO 2022 by H. Burnett et al. demonstrated that fruquintinib had the longest median progression-free survival (mPFS) and the highest reduction in the risk of disease progression or death among all currently approved third-line treatments for mCRC. Additionally, regorafenib 80+ showed superior overall survival (OS) compared to other treatment options, in line with our study results (28). However, due to the lack of comparison and analysis with the combination of TAS-102 and bevacizumab in these studies, we included this treatment option in our analysis for the first time. This allowed us to more accurately assess and optimize third-line treatment options through systematic review and NMA, offering guidance for selecting appropriate treatments for patients with mCRC.

Based on our research analysis, TAS-102 plus bevacizumab emerged as the most effective treatment in terms of both OS and PFS among all the included options, followed by regorafenib 80+ and fruquintinib. TAS-102 has demonstrated antitumor activity against fluorouracil-resistant cell lines in preclinical xenograft models, which has important implications for CRC treatment (29, 30). Bevacizumab is a recombinant humanized immunoglobulin G1 (IgG1) monoclonal antibody that inhibits the binding of VEGF-A to VEGF receptor-2 (VEGFR-2). It can also modulate the immune system of CRC patients by inhibiting the maturation of tumor microenvironment (TME) dendritic cells (31). The combination of bevacizumab with TAS-102 may enhance the accumulation and phosphorylation levels of trifluorothymidine in tumor DNA without increasing systemic exposure or toxicity, thereby improving treatment efficacy (32).

In terms of adverse events (AEs), regorafenib exhibits a higher toxicity profile compared to other regimens. Common ≥3AEs include hand-foot syndrome (HFS), fatigue, and hypertension (20–22). Studies have shown that regorafenib-related AEs are dose-dependent, primarily occurring in the initial treatment cycles. In an effort to mitigate regorafenib toxicity, the REDOS study explored a dose escalation strategy to prolong the duration of treatment as tolerated by patients. The results demonstrated that treatment efficacy was not compromised in the dose-escalation group compared to the standard dose group, and the incidence of AEs was relatively low. Patients in the dose-escalation group also reported slightly higher overall quality of life (QOL) scores on the questionnaires, although the difference was not significant (22). However, due to the small sample size of this study, further research is needed to investigate the dosing aspects of regorafenib. The most common AEs associated with fruquintinib were hypertension, HFS, and proteinuria, similar to regorafenib but with much less fruquintinib toxicity across all classes of toxicity (13). The three regimens mentioned above generally have less hematological toxicity compared to TAS-102 (11, 12, 23). TAS-102 plus bevacizumab exhibits similar AEs to TAS-102 alone, with a higher incidence of severe neutropenia but no increased incidence of febrile neutropenia. These AEs are manageable (23, 25). Therefore, the choice of an appropriate treatment regimen can be based on the AEs associated with each option, taking into consideration the patient’s individual condition.

Compared to other treatment protocols within the same subgroups, TAS-102 plus bevacizumab demonstrated the greatest improvement in survival among patients aged 65 years or older, female patients, and patients with a time of 18 months or more from the first diagnosis of metastatic disease to randomization. The SUNLIGHT study also demonstrated the efficacy of TAS-102 plus bevacizumab across different RAS mutation statuses (25). RAS mutations upregulate VEGF expression, promoting tumor angiogenesis in CRC, while bevacizumab effectively inhibits VEGF activity, delaying tumor growth and metastasis. The mechanism of action of TAS-102 involves the direct binding of FTD to DNA, indicating that RAS mutations do not directly affect the activity of TAS-102 plus bevacizumab (9, 33). The order of drug use can also impact treatment effectiveness, as demonstrated in the REVERCE study, a phase II clinical trial comparing two treatment sequences in patients with KRAS wild-type mCRC. The results showed that the regimen of regorafenib followed by cetuximab was superior to the regimen of cetuximab followed by regorafenib in terms of mOS (17.4 months *vs*. 11.6 months, P = 0.0293). This suggests that using regorafenib as the initial treatment may enhance the survival benefits for patients (34). Similar findings were observed in the RESOURCE trial, where patients previously treated with regorafenib maintained a longer survival benefit when retreated with TAS-102 (11). Notably, the CONCUR study reported a significantly greater OS benefit compared to the CORRECT study, which may be partly attributed to the inclusion of patients who had not received targeted therapy in the CONCUR trial, while the patients in the CORRECT study had received at least one targeted biological drug treatment (20, 21). These findings further support the consideration of early utilization of regorafenib.

In addition to the studies analyzed in this paper, there are other treatments worth considering. For instance, a meta-analysis conducted by Thomas Walter et al. on third-line treatment for mCRC included studies on selective internal radiation therapy (SIRT), which demonstrated that SIRT resulted in greater OS benefits for patients with liver metastases compared to systemic therapy while reducing the incidence of toxicity (26). Patients with mCRC with high microsatellite instability (MSI-H) or defective mismatch repair (dMMR) have shown better survival rates compared to those with microsatellite stability (MSS) or low microsatellite instability (MSI-L), and they have exhibited greater sensitivity to immune checkpoint inhibitor therapy (35, 36). In the REGONIVO study, the combination of regorafenib and nivolumab showed promising efficacy in MSS mCRC, with an objective remission rate (ORR) of 36% and a median progression-free survival (mPFS) of 7.9 months. This combination regimen demonstrated superior efficacy compared to regorafenib or nivolumab monotherapy, although the study had a small sample size and further validation is needed (37). Approximately 8–10% of mCRC patients have BRAF mutations, with over 90% of these mutations occurring at the V600E locus (38). In the randomized phase III BEACON study, encorafenib plus cetuximab, with or without binimetinib, showed longer OS and higher response rates compared to standard therapy (irinotecan or FOLFIRI and cetuximab) in patients with BRAF V600E-mutated mCRC who had received prior treatment. Based on the BEACON study, encorafenib in combination with cetuximab was approved by the FDA in 2020 for the treatment of patients with BRAF V600E-mutated mCRC (39).

There are several limitations to this study. Firstly, the number of clinical studies we included and the sample size of patients were limited. Furthermore, some of the included studies had inconsistent or incomplete content for subgroup analysis, which resulted in insufficient research results. Additionally, some of the definitions of AEs differed between the RCTs included in this study, which may have led to inconsistent findings. Moreover, our NMA was unable to create a closed loop, so no Bayesian method of nodal analysis or direct element analysis by the frequency method was performed. Therefore, we were unable to assess inconsistencies in the analysis due to heterogeneity (40). Although this NMA focused on third-line treatment studies, trials involving first-line, second-line, or more lines of treatment for patients were also included in the analysis. It is worth noting that different studies have different inclusion criteria, and ethnic differences in patients included in different studies may also lead to biased results. Therefore, we hope that more third-line studies of patients with mCRC can be conducted in multiple centers worldwide, enabling direct comparison of the efficacy of different treatment regimens and detailed analysis for different subgroups, in order to provide guidance for the development of precise, individualized treatment plans for patients.

## Conclusions

Based on the results of the analysis of treatment efficacy, safety, and subgroups in this study, it was found that regorafenib and TAS-102 had similar efficacy. However, regorafenib had the highest toxicity compared to other treatment options. TAS-102 combined with bevacizumab may be the optimal third-line therapy for patients with mCRC compared to the other treatment options included in this study. However, due to the limitations of the included studies in terms of number and quality, these results should be further confirmed by large-scale RCTs in the future.

## Data availability statement

The original contributions presented in the study are included in the article/**Supplementary Material**. Further inquiries can be directed to the corresponding author.

## Author contributions

LG: Data curation, Formal Analysis, Investigation, Software, Writing – original draft, Writing – review & editing. LT: Data curation, Formal Analysis, Investigation, Methodology, Writing – review & editing. ZH: Data curation, Formal Analysis, Investigation, Writing – review & editing. JP: Formal Analysis, Investigation, Methodology, Writing – review & editing. XL: Conceptualization, Formal Analysis, Investigation, Methodology, Writing – review & editing. BL: Conceptualization, Formal Analysis, Methodology, Supervision, Writing – review & editing.

## References

[1] Torre LA Bray F Siegel RL Ferlay J Lortet-Tieulent J Jemal A . Global cancer statistics, 2012. CA Cancer J Clin (2015) 65(2):87–108. doi: 10.3322/caac.21262 25651787

[2] Dekker E Tanis PJ Vleugels JLA Kasi PM Wallace MB . Colorectal cancer. Lancet. (2019) 394(10207):1467–80. doi: 10.1016/S0140-6736(19)32319-0 31631858

[3] Böckelman C Engelmann BE Kaprio T Hansen TF Glimelius B . Risk of recurrence in patients with colon cancer stage II and III: a systematic review and meta-analysis of recent literature. Acta Oncol (2015) 54(1):5–16. doi: 10.3109/0284186X.2014.975839 25430983

[4] Siegel RL Miller KD Jemal A . Cancer statistics, 2019. CA Cancer J Clin (2019) 69(1):7–34. doi: 10.3322/caac.21551 30620402

[5] Cervantes A Adam R Roselló S Arnold D Normanno N Taïeb J . Metastatic colorectal cancer: ESMO Clinical Practice Guideline for diagnosis, treatment and follow-up. Ann Oncol (2023) 34(1):10–32. doi: 10.1016/j.annonc.2022.10.003 36307056

[6] Benson AB Venook AP Al-Hawary MM Arain MA Chen YJ Ciombor KK . Colon cancer, version 2.2021, NCCN clinical practice guidelines in oncology. J Natl Compr Canc Netw (2021) 19(3):329–59. doi: 10.6004/jnccn.2021.0012 33724754

[7] Wilhelm SM Dumas J Adnane L . Regorafenib (BAY 73-4506): a new oral multikinase inhibitor of angiogenic, stromal and oncogenic receptor tyrosine kinases with potent preclinical antitumor activity. Int J Cancer. (2011) 129(1):245–55. doi: 10.1002/ijc.25864\ 21170960

[8] van der Velden DL Opdam FL Opdam FL . TAS-102 and the quest for predictive biomarkers. ESMO Open (2017) 2(4):e000263. doi: 10.1136/esmoopen-2017-000263 29018579 PMC5623337

[9] Tanaka N Sakamoto K Okabe H Fujioka A Yamamura K Nakagawa F . Repeated oral dosing of TAS-102 confers high trifluridine incorporation into DNA and sustained antitumor activity in mouse models. Oncol Rep (2014) 32(6):2319–26. doi: 10.3892/or.2014.3487 PMC4240496 25230742

[10] Fukushima M Suzuki N Emura T Yano S Kazuno H Tada Y . Structure and activity of specific inhibitors of thymidine phosphorylase to potentiate the function of antitumor 2’-deoxyribonucleosides. Biochem Pharmacol (2000) 59(10):1227–36. doi: 10.1016/s0006-2952(00)00253-7 10736423

[11] Mayer RJ Van Cutsem E Falcone A Yoshino T Garcia-Carbonero R Mizunuma N . Randomized trial of TAS-102 for refractory metastatic colorectal cancer. N Engl J Med (2015) 372(20):1909–19. doi: 10.1056/NEJMoa1414325 25970050

[12] Xu J Kim TW Shen L Sriuranpong V Pan H Xu R . Results of a randomized, double-blind, placebo-controlled, phase III trial of trifluridine/tipiracil (TAS-102) monotherapy in asian patients with previously treated metastatic colorectal cancer: the TERRA study. J Clin Oncol (2018) 36(4):350–8. doi: 10.1200/JCO.2017.74.3245 29215955

[13] Li J Qin S Xu RH Shen L Xu J Bai Y . Effect of fruquintinib vs placebo on overall survival in patients with previously treated metastatic colorectal cancer: the FRESCO randomized clinical trial. JAMA. (2018) 319(24):2486–96. doi: 10.1001/jama.2018.7855 PMC6583690 29946728

[14] Hutton B Salanti G Caldwell DM Chaimani A Schmid CH Cameron C . The PRISMA extension statement for reporting of systematic reviews incorporating network meta-analyses of health care interventions: checklist and explanations. Ann Intern Med (2015) 162(11):777–84. doi: 10.7326/M14-2385 26030634

[15] Higgins JP Thompson SG Deeks JJ Altman DG . Measuring inconsistency in meta-analyses. BMJ. (2003) 327(7414):557–60. doi: 10.1136/bmj.327.7414.557 PMC192859 12958120

[16] Dias S Welton NJ Caldwell DM Ades AE . Checking consistency in mixed treatment comparison meta-analysis. Stat Med (2010) 29(7-8):932–44. doi: 10.1002/sim.3767 20213715

[17] Neupane B Richer D Bonner AJ Kibret T Beyene J . Network meta-analysis using R: a review of currently available automated packages. PloS One (2014) 9(12):e115065. doi: 10.1371/journal.pone.0115065 25541687 PMC4277278

[18] Brooks SP Gelman A . General methods for monitoring convergence of iterative simulations. J Comput Graphical Stat (1998) 7(4):434–55. doi: 10.1080/10618600.1998.10474787

[19] Salanti G Ades AE Ioannidis JP . Graphical methods and numerical summaries for presenting results from multiple-treatment meta-analysis: an overview and tutorial. J Clin Epidemiol. (2011) 64(2):163–71. doi: 10.1016/j.jclinepi.2010.03.016 20688472

[20] Grothey A Van Cutsem E Sobrero A Siena S Falcone A Ychou M . Regorafenib monotherapy for previously treated metastatic colorectal cancer (CORRECT): an international, multicentre, randomised, placebo-controlled, phase 3 trial. Lancet. (2013) 381(9863):303–12. doi: 10.1016/S0140-6736(12)61900-X 23177514

[21] Li J Qin S Xu R Yau TC Ma B Pan H . Regorafenib plus best supportive care versus placebo plus best supportive care in Asian patients with previously treated metastatic colorectal cancer (CONCUR): a randomised, double-blind, placebo-controlled, phase 3 trial. Lancet Oncol (2015) 16(6):619–29. doi: 10.1016/S1470-2045(15)70156-7 25981818

[22] Bekaii-Saab TS Ou FS Ahn DH Boland PM Ciombor KK Heying EN . Regorafenib dose-optimisation in patients with refractory metastatic colorectal cancer (ReDOS): a randomised, multicentre, open-label, phase 2 study. Lancet Oncol (2019) 20(8):1070–82. doi: 10.1016/S1470-2045(19)30272-4 PMC9187307 31262657

[23] Yoshino T Mizunuma N Yamazaki K Nishina T Komatsu Y Baba H . TAS-102 monotherapy for pretreated metastatic colorectal cancer: a double-blind, randomised, placebo-controlled phase 2 trial. Lancet Oncol (2012) 13(10):993–1001. doi: 10.1016/S1470-2045(12)70345-5 22951287

[24] Pfeiffer P Yilmaz M Möller S Zitnjak D Krogh M Petersen LN . TAS-102 with or without bevacizumab in patients with chemorefractory metastatic colorectal cancer: an investigator-initiated, open-label, randomised, phase 2 trial. Lancet Oncol (2020) 21(3):412–20. doi: 10.1016/S1470-2045(19)30827-7 31999946

[25] Prager GW Taieb J Fakih M Ciardiello F Van Cutsem E Elez E . Trifluridine-tipiracil and bevacizumab in refractory metastatic colorectal cancer. N Engl J Med (2023) 388(18):1657–67. doi: 10.1056/NEJMoa2214963 37133585

[26] Walter T Hawkins NS Pollock RF Colaone F Shergill S Ross PJ . Systematic review and network meta-analyses of third-line treatments for metastatic colorectal cancer. J Cancer Res Clin Oncol (2020) 146(10):2575–87. doi: 10.1007/s00432-020-03315-6 PMC7467965 32715436

[27] Sonbol MB Benkhadra R Wang Z Firwana B Walden DJ Mody K . A systematic review and network meta-analysis of regorafenib and TAS-102 in refractory metastatic colorectal cancer. Oncologist. (2019) 24(9):1174–9. doi: 10.1634/theoncologist.2019-0189 PMC6738316 31164455

[28] Burnett H Proskorovsky I Yoon SS Wang Y Ostojic H Gaianu L . 400P Impact of regorafenib dose optimization on comparative outcomes in the treatment of relapsed/refractory metastatic colorectal cancer (mCRC)[J]. Ann Oncol (2022) 33:S719–20. doi: 10.1016/j.annonc.2022.07.538

[29] Emura T Murakami Y Nakagawa F Fukushima M Kitazato K . A novel antimetabolite, TAS-102 retains its effect on FU-related resistant cancer cells. Int J Mol Med (2004) 13(4):545–9. doi: 10.3892/ijmm.13.4.545 15010854

[30] Emura T Suzuki N Yamaguchi M Ohshimo H Fukushima M . A novel combination antimetabolite, TAS-102, exhibits antitumor activity in FU-resistant human cancer cells through a mechanism involving FTD incorporation in DNA. Int J Oncol (2004) 25(3):571–8. doi: 10.3892/ijo.25.3.571 15289858

[31] Michielsen AJ Noonan S Martin P Tosetto M Marry J Biniecka M . Inhibition of dendritic cell maturation by the tumor microenvironment correlates with the survival of colorectal cancer patients following bevacizumab treatment. Mol Cancer Ther (2012) 11(8):1829–37. doi: 10.1158/1535-7163.MCT-12-0162 22675042

[32] Kuboki Y Nishina T Shinozaki E Yamazaki K Shitara K Okamoto W . TAS-102 plus bevacizumab for patients with metastatic colorectal cancer refractory to standard therapies (C-TASK FORCE): an investigator-initiated, open-label, single-arm, multicentre, phase 1/2 study. Lancet Oncol (2017) 18(9):1172–81. doi: 10.1016/S1470-2045(17)30425-4 28760399

[33] Rak J Mitsuhashi Y Bayko L Filmus J Shirasawa S Sasazuki T . Mutant ras oncogenes upregulate VEGF/VPF expression: implications for induction and inhibition of tumor angiogenesis. Cancer Res (1995) 55(20):4575–80. 7553632

[34] Shitara K Yamanaka T Denda T Tsuji Y Shinozaki K Komatsu Y . REVERCE: a randomized phase II study of regorafenib followed by cetuximab versus the reverse sequence for previously treated metastatic colorectal cancer patients. Ann Oncol (2019) 30(2):259–65. doi: 10.1093/annonc/mdy526 30508156

[35] Le DT Uram JN Wang H Bartlett BR Kemberling H Eyring AD . PD-1 blockade in tumors with mismatch-repair deficiency. N Engl J Med (2015) 372(26):2509–20. doi: 10.1056/NEJMoa1500596 PMC4481136 26028255

[36] Overman MJ McDermott R Leach JL Lonardi S Lenz HJ Morse MA . Nivolumab in patients with metastatic DNA mismatch repair-deficient or microsatellite instability-high colorectal cancer (CheckMate 142): an open-label, multicentre, phase 2 study. Lancet Oncol (2017) 18(9):1182–91. doi: 10.1016/S1470-2045(17)30422-9 PMC6207072 28734759

[37] Fukuoka S Hara H Takahashi N Kojima T Kawazoe A Asayama M . Regorafenib plus nivolumab in patients with advanced gastric or colorectal cancer: an open-label, dose-escalation, and dose-expansion phase ib trial (REGONIVO, EPOC1603). J Clin Oncol (2020) 38(18):2053–61. doi: 10.1200/JCO.19.03296 32343640

[38] Garrido-Ramos MA . Satellite DNA: an evolving topic. Genes (2017) 8(9):230. doi: 10.3390/genes8090230 28926993 PMC5615363

[39] Kopetz S Grothey A Yaeger R Van Cutsem E Desai J Yoshino T . Encorafenib, binimetinib, and cetuximab in *BRAF* V600E-mutated colorectal cancer. N Engl J Med (2019) 381(17):1632–43. doi: 10.1056/NEJMoa1908075 31566309

[40] Rouse B Chaimani A Li T . Network meta-analysis: an introduction for clinicians. Intern Emerg Med (2017) 12(1):103–11. doi: 10.1007/s11739-016-1583-7 PMC5247317 27913917

