# Supplementary material for: Comparison of the efficacy and safety of third-line treatments for metastatic colorectal cancer: a systematic review and network meta-analysis
Source: Front Oncol. 2023 Sep 21;13:1269203. doi: 10.3389/fonc.2023.1269203 (PMC10552753; doi:10.3389/fonc.2023.1269203)
Supplement: Supplementary file 1 [file DataSheet_1.docx]

***Supplementary Material***

**Supplementary Table 1. Checklist of the PRISMA extension for network meta-analysis.**

| *Section/topic* | *Item#* | *Checklist item* |
| --- | --- | --- |
| TITLE | | |
| Title | 1 | Identify the report as a systematic review incorporating a network meta- analysis (or related form of meta-analysis). |
| ABSTRACT | | |
| Structured summary | 2 | Provide a structured summary including, as applicable:  •Background: main objectives;  • Methods: data sources; study eligibility criteria, participants, and interventions; study appraisal and synthesis methods, such as network meta- analysis. • Results: number of studies and participants identified; summary estimates with corresponding confidence/credible intervals; treatment rankings may also be discussed. Authors may choose to summarize pairwise comparisons against a chosen treatment included in their analyses for brevity. • Discussion/Conclusions: limitations; conclusions and implications of findings.  • Other: primary source of funding; systematic review registration number with registry name. |
| INTRODUCTION | | |
| Rationale | 3 | Describe the rationale for the review in the context of what is already known, including mention of why a network meta-analysis has been conducted. |
| Objectives | 4 | Provide an explicit statement of questions being addressed with reference to participants, interventions, comparisons, outcomes, and study design (PICOS). |
| METHODS | | |
| Protocol and registration | 5 | Indicate if a review protocol exists, if and where it can be accessed (e.g., Web address), and, if available, provide registration information including registration number. |
| Eligibility criteria | 6 | Specify study characteristics (e.g., PICOS, length of follow-up) and report characteristics (e.g., years considered, language, publication status) used as criteria for eligibility, giving rationale. Clearly describe eligible treatments included in the treatment network, and note whether any have been clusteredor merged into the same node (with justification). |
| Information sources | 7 | Describe all information sources (e.g., databases with dates of coverage, contact with study authors to identify additional studies) in the search and date last searched. |
| Search | 8 | Present full electronic search strategy for at least one database, including any limits used, such that it could be repeated. |
| Study selection | 9 | State the process for selecting studies (i.e., screening, eligibility, included in systematic review, and, if applicable, included in the meta-analysis). |
| Data collection process | 10 | Describe method of data extraction from reports (e.g., piloted forms, independently, in duplicate) and any processes for obtaining and confirming data from investigators. |
| Data items | 11 | List and define all variables for which data were sought (e.g., PICOS, funding sources) and any assumptions and simplifications made. |
| Geometry of the network | S1 | Describe methods used to explore the geometry of the treatment network under study and potential biases related to it. This should include how the evidence base has been graphically summarized for presentation, and what characteristics were compiled and used to describe the evidence base to readers. |
| Risk of bias in individual studies | 12 | Describe methods used for assessing risk of bias of individual studies (including specification of whether this was done at the study or outcome level), and how this information is to be used in any data synthesis. |
| Summary measures | 13 | State the principal summary measures (e.g., risk ratio, difference in means). Also describe the use of additional summary measures assessed, such as treatment rankings and surface under the cumulative ranking curve (SUCRA) values, as well as modified approaches used to present summary findings from meta-analyses. |
| Synthesis of results | 14 | Describe the methods of handling data and combining results of studies for each network meta-analysis. This should include, but not be limited to:  • Handling of multi-arm trials;  • Selection of variance structure;  • Selection of prior distributions in Bayesian analyses; and  • Assessment of model fit. |
| Assessment of Inconsistency | S2 | Describe the statistical methods used to evaluate the agreement of direct and indirect evidence in the treatment network(s) studied. Describe efforts taken to address its presence when found. |
| Risk of bias across studies | 15 | Specify any assessment of risk of bias that may affect the cumulative evidence (e.g., publication bias, selective reporting within studies). |
| Additional analyses | 16 | Describe methods of additional analyses, if done, indicating which were pre- specified. This may include, but not be limited to the following:  • Sensitivity or subgroup analyses;  • Meta-regression analyses;  •Alternative formulations of the treatment network; and  • Use of alternative prior distributions for Bayesian analyses (if applicable). |
| RESULTS | | |
| Study selection | 17 | Give numbers of studies screened, assessed for eligibility, and included in the review, with reasons for exclusions at each stage, ideally with a flow diagram. |
| Presentation of Network structure | S3 | Provide a network graph of the included studies to enable visualization of the geometry of the treatment network |
| Summary of network geometry | S4 | Provide a brief overview of characteristics of the treatment network. This may include commentary on the abundance of trials and randomized patients for the different interventions and pairwise comparisons in the network, gaps of evidence in the treatment network, and potential biases reflected by the network structure. |
| Study characteristics | 18 | For each study, present characteristics for which data were extracted (e.g., study size, PICOS, follow-up period) and provide the citations. |
| Risk of bias within studies | 19 | Present data on risk of bias of each study and, if available, any outcome level assessment. |
| Results of individual studies | 20 | For all outcomes considered (benefits or harms), present, for each study:1) simple summary data for each intervention group, and 2) effect estimates and confidence/credible intervals. Modified approaches may be needed to deal with information from larger networks. |
| Synthesis of results | 21 | Present results of each meta-analysis done, including confidence/credible intervals. In larger networks, authors may focus on comparisons versus a particular comparator (e.g. placebo or standard care), with full findings presented in an appendix. League tables and forest plots may be considered to summarize pairwise comparisons. If additional summary measures were explored (such as treatment rankings), these should also be presented. |
| Exploration for inconsistency | S5 | Describe results from investigations of inconsistency. This may include such information as measures of model fit to compare consistency and inconsistency models, P values from statistical tests, or summary of inconsistency estimates from different parts of the treatment network. |
| Risk of bias across studies | 22 | Present results of any assessment of risk of bias across studies. |
| Additional analysis | 23 | Give results of additional analyses, if done (e.g., sensitivity or subgroup analyses, meta-regression, alternative network geometries studied, alternative choice of prior distributions for Bayesian analyses, and so forth. |
| DISCUSSION | | |
| Summary of evidence | 24 | Summarize the main findings including the strength of evidence for each main outcome; consider their relevance to key groups (e.g., healthcare providers, users, and policy makers). |
| Limitations | 25 | Discuss limitations at study and outcome level (e.g., risk of bias), and at review-level (e.g., incomplete retrieval of identified research, reporting bias). Comment on the validity of the assumptions, such as transitivity and consistency. Comment on any concerns regarding network geometry (e.g., avoidance of certain comparisons). |
| Conclusions | 26 | Provide a general interpretation of the results in the context of other evidence, and implications for future research. |
| FUNDING | 27 | Describe sources of funding for the systematic review and other support (e.g., supply of data); role of funders for the systematic review. |

**Supplementary Table 2. Literature search criteria.**

**A. Search strategy on Pubmed.**

(((((((((((((((("Colorectal Neoplasms"[Mesh]) OR (Colorectal Neoplasm[Title/Abstract])) OR (Neoplasm, Colorectal[Title/Abstract])) OR (Neoplasms, Colorectal[Title/Abstract])) OR (Neoplasms, Colorectal[Title/Abstract])) OR (Colorectal Tumor[Title/Abstract])) OR (Tumor, Colorectal[Title/Abstract])) OR (Tumors, Colorectal[Title/Abstract])) OR (Colorectal Cancer[Title/Abstract])) OR (Cancer, Colorectal[Title/Abstract])) OR (Cancers, Colorectal[Title/Abstract])) OR (Colorectal Cancers[Title/Abstract])) OR (Colorectal Carcinoma[Title/Abstract])) OR (Carcinoma, Colorectal[Title/Abstract])) OR (Carcinomas, Colorectal[Title/Abstract])) OR (Colorectal Carcinomas[Title/Abstract])) AND (((((((((((((((((((((((((((((((((TAS102) OR (trifluridine - tipiracil[Title/Abstract])) OR (trifluridine[Title/Abstract] AND tipiracil[Title/Abstract])) OR (trifluridine tipiracil[Title/Abstract])) OR (TAS 102[Title/Abstract])) OR (TAS-102[Title/Abstract])) OR (Lonsur[Title/Abstract])) OR (Fruquintinib[Title/Abstract])) OR (Regorafenib[Title/Abstract])) OR (4-(4-(((4-chloro-3-(trifluoromethyl)phenyl)carbamoyl)amino)-3-fluorophenoxy)-n-methylpyridine-2-carboxamide[Title/Abstract])) OR (Stivarga[Title/Abstract])) OR (BAY 73-4506[Title/Abstract])) OR (BAY73-4506[Title/Abstract])) OR (BAY-73-4506[Title/Abstract])) OR (Anlotinib[Title/Abstract])) OR (AL3818[Title/Abstract])) OR (Raltitrexed[Title/Abstract])) OR (N-(5-(N-(3,4-dihydro-2-methyl-4-oxoquinazolin-6-ylmethyl)-N-methylamino)-2-thenoyl)-L-glutamic acid[Title/Abstract])) OR (ICI D1694[Title/Abstract])) OR (ICI-D1694[Title/Abstract])) OR (ZD 1694[Title/Abstract])) OR (ZD1694[Title/Abstract])) OR (ZD-1694[Title/Abstract])) OR (D1694[Title/Abstract])) OR (D 1694[Title/Abstract])) OR (D-1694[Title/Abstract])) OR (Tomudex[Title/Abstract])) OR (VEGF[Title/Abstract])) OR (EGFR[Title/Abstract])) OR (((((("Cetuximab"[Mesh]) OR (Erbitux[Title/Abstract])) OR (IMC C225[Title/Abstract])) OR (IMC-C225[Title/Abstract])) OR (MAb C225[Title/Abstract])) OR (C225[Title/Abstract]))) OR (((((((((((((((("Irinotecan"[Mesh]) OR (Camptothecin-11[Title/Abstract])) OR (Camptothecin 11[Title/Abstract])) OR (SN 38 11[Title/Abstract])) OR (SN-38-11[Title/Abstract])) OR (SN3811[Title/Abstract])) OR (SN 38[Title/Abstract])) OR (SN-38[Title/Abstract])) OR (NK012 Compound[Title/Abstract])) OR (CPT-11[Title/Abstract])) OR (CPT11[Title/Abstract])) OR (CPT 11[Title/Abstract])) OR (Camptosar[Title/Abstract])) OR (7-Ethyl-10-hydroxycamptothecin[Title/Abstract])) OR (7 Ethyl 10 hydroxycamptothecin[Title/Abstract])) OR (Irinotecan Hydrochloride[Title/Abstract]))) OR ((((("Bevacizumab"[Mesh]) OR (Mvasi[Title/Abstract])) OR (Bevacizumab-awwb[Title/Abstract])) OR (Bevacizumab awwb[Title/Abstract])) OR (Avastin[Title/Abstract]))) OR (((((((("Drug Therapy"[Mesh]) OR (Therapy, Drug[Title/Abstract])) OR (Drug Therapies[Title/Abstract])) OR (Therapies, Drug[Title/Abstract])) OR (Chemotherapies[Title/Abstract])) OR (Pharmacotherapy[Title/Abstract])) OR (Pharmacotherapies[Title/Abstract])) OR (Chemotherapy[Title/Abstract]))) AND (((((((((((3L[Title/Abstract]) OR (3 line[Title/Abstract])) OR (third line[Title/Abstract])) OR (third-line[Title/Abstract])) OR (3-line[Title/Abstract])) OR (Previously Treated[Title/Abstract])) OR (3-L[Title/Abstract])) OR (Treated[Title/Abstract])) OR (Later Treated[Title/Abstract])) OR (pretreated[Title/Abstract])) OR (placebo[Title/Abstract])) Filters: Clinical Trial, from 2005 - 2023

**B. Search strategy on Embase.**

#1 ('colorectal neoplasms':ab,ti OR 'colorectal neoplasm':ab,ti OR 'neoplasm, colorectal':ab,ti OR 'neoplasms, colorectal':ab,ti OR 'colorectal tumors':ab,ti OR 'colorectal tumor':ab,ti OR 'tumor, colorectal':ab,ti OR 'tumors, colorectal':ab,ti OR 'colorectal cancer':ab,ti OR 'cancer, colorectal':ab,ti OR 'cancers, colorectal':ab,ti OR 'colorectal cancers':ab,ti OR 'colorectal carcinoma':ab,ti OR 'carcinoma, colorectal':ab,ti OR 'carcinomas, colorectal':ab,ti OR 'colorectal carcinomas':ab,ti) AND ([controlled clinical trial]/lim OR [randomized controlled trial]/lim) AND [2005-2023]/py

#2 (tas102:ab,ti OR 'trifluridine - tipiracil':ab,ti OR trifluridine:ab,ti OR tipiracil:ab,ti OR 'trifluridine tipiracil':ab,ti OR 'tas 102':ab,ti OR lonsur:ab,ti OR fruquintinib:ab,ti OR regorafenib:ab,ti OR '4 4 4 chloro 3 trifluoromethylphenylcarbamoylamino 3 fluorophenoxy n methylpyridine 2 carboxamide':ab,ti OR stivarga:ab,ti OR 'bay 73-4506':ab,ti OR 'bay73 4506':ab,ti OR 'bay 73 4506':ab,ti OR anlotinib:ab,ti OR al3818:ab,ti OR raltitrexed:ab,ti OR 'n-5-n-3,4-dihydro-2-methyl-4-oxoquinazolin-6-ylmethyl-n-methylamino-2-thenoyl-l-glutamic acid':ab,ti OR 'ici d1694':ab,ti OR zd1694:ab,ti OR 'zd 1694':ab,ti OR d1694:ab,ti OR 'd 1694':ab,ti OR tomudex:ab,ti OR cetuximab:ab,ti OR erbitux:ab,ti OR 'imc c225':ab,ti OR 'mab c225':ab,ti OR c225:ab,ti OR irinotecan:ab,ti OR 'camptothecin 11':ab,ti OR 'sn 38 11':ab,ti OR sn3811:ab,ti OR 'sn 38':ab,ti OR 'nk012 compound':ab,ti OR cpt11:ab,ti OR 'cpt 11':ab,ti OR camptosar:ab,ti OR '7 ethyl 10 hydroxycamptothecin':ab,ti OR 'irinotecan hydrochloride':ab,ti OR bevacizumab:ab,ti OR mvasi:ab,ti OR 'bevacizumab awwb':ab,ti OR avastin:ab,ti OR 'drug therapy':ab,ti OR 'therapy, drug':ab,ti OR 'drug therapies':ab,ti OR 'therapies, drug':ab,ti OR chemotherapies:ab,ti OR pharmacotherapy:ab,ti OR pharmacotherapies:ab,ti OR chemotherapy:ab,ti OR vegf:ab,ti OR egfr:ab,ti) AND ([controlled clinical trial]/lim OR [randomized controlled trial]/lim) AND [2005-2023]/py

#3 (3l:ab,ti OR '3 line':ab,ti OR 'third line':ab,ti OR 'previously treated':ab,ti OR '3 l':ab,ti OR treated:ab,ti OR 'later treated':ab,ti OR pretreated:ab,ti OR placebo:ab,ti) AND ([controlled clinical trial]/lim OR [randomized controlled trial]/lim) AND [2005-2023]/py

#4 #1 AND #2 AND #3

**C. Search strategy on Web of science.**

TS=(Colorectal Neoplasms OR Colorectal Neoplasm OR Neoplasm, Colorectal OR Neoplasms, Colorectal OR Colorectal Tumors OR Colorectal Tumor OR Tumor, Colorectal OR Tumors, Colorectal OR Colorectal Cancer OR Cancer, Colorectal OR Cancers, Colorectal OR Colorectal Cancers OR Colorectal Carcinoma OR Carcinoma, Colorectal OR Carcinomas, Colorectal OR Colorectal Carcinomas) AND TS=( TAS102 OR trifluridine - tipiracil OR trifluridine OR tipiracil OR trifluridine tipiracil OR TAS 102 OR TAS-102 OR Lonsur OR Fruquintinib OR Regorafenib OR 4-4-4-chloro-3-trifluoromethylphenylcarbamoylamino-3-fluorophenoxy-n-methylpyridine-2-carboxamide OR Stivarga OR BAY 73-4506 OR BAY73-4506 OR BAY-73-4506 OR Anlotinib OR AL3818 OR Raltitrexed OR N-5-N-3,4-dihydro-2-methyl-4-oxoquinazolin-6-ylmethyl-N-methylamino-2-thenoyl-L-glutamic acid OR ICI D1694 OR ICI-D1694 OR ZD 1694 OR ZD1694 OR ZD-1694 OR D1694 OR D 1694 OR D-1694 OR Tomudex OR VEGF OR EGFR OR Cetuximab OR Erbitux OR IMC C225 OR IMC-C225 OR MAb C225 OR C225 OR Irinotecan OR Camptothecin-11 OR Camptothecin 11 OR SN 38 11 OR SN-38-11 OR SN3811 OR SN 38 OR SN-38 OR NK012 Compound OR CPT-11 OR CPT11 OR CPT 11 OR Camptosar OR 7-Ethyl-10-hydroxycamptothecin OR 7 Ethyl 10 hydroxycamptothecin OR Irinotecan Hydrochloride OR Bevacizumab OR Mvasi OR Bevacizumab-awwb OR Bevacizumab awwb OR Avastin OR Drug Therapy OR Therapy, Drug OR Drug Therapies OR Therapies, Drug OR Chemotherapies OR Pharmacotherapy OR Pharmacotherapies OR Chemotherapy OR VEGF OR EGFR) AND TS=(3L OR 3 line OR 3-line OR third line OR third-line OR Previously Treated OR 3-L OR Treated OR Later Treated OR pretreated OR placebo)

**D. Search strategy on Cochrane Central Register of Controlled Trials.**

#1 MeSH descriptor: [Colorectal Neoplasms] explode all trees

#2 (Colorectal Neoplasm OR Neoplasm, Colorectal OR Neoplasms, Colorectal OR Colorectal Tumors OR Colorectal Tumor OR Tumor, Colorectal OR Tumors, Colorectal OR Colorectal Cancer OR Cancer, Colorectal OR Cancers, Colorectal OR Colorectal Cancers OR Colorectal Carcinoma OR Carcinoma, Colorectal OR Carcinomas, Colorectal OR Colorectal Carcinomas): ti,ab,kw (Word variations have been searched)

#3 #1 or #2

#4 TAS102 OR trifluridine-tipiracil OR trifluridine OR tipiracil OR trifluridine tipiracil OR TAS 102 OR TAS-102 OR Lonsur OR Fruquintinib OR Regorafenib OR Anlotinib OR AL3818 OR Raltitrexed OR ICI D1694 OR ICI-D1694 OR ZD 1694 OR ZD1694 OR ZD-1694 OR D1694 OR D 1694 OR D-1694 OR Tomudex OR VEGF OR EGFR

#5 MeSH descriptor: [Cetuximab] explode all trees

#6 Erbitux OR IMC C225 OR IMC-C225 OR MAb C225 OR C225

#7 #5 or #6

#8 MeSH descriptor: [Irinotecan] explode all trees

#9 Irinotecan Camptothecin-11 OR Camptothecin 11 OR SN 38 11 OR SN3811 OR SN 38 OR SN-38 OR NK012 Compound OR CPT-11 OR CPT11 OR CPT 11 OR Camptosar OR 7 Ethyl 10 hydroxycamptothecin OR Irinotecan Hydrochloride

#10 #8 or #9

#11 MeSH descriptor: [Bevacizumab] explode all trees

#12 Mvasi OR Bevacizumab-awwb OR Bevacizumab awwb OR Avastin

#13 #11 or #12

#14 MeSH descriptor: [Drug Therapy] explode all trees

#15 Therapy, Drug OR Drug Therapies OR Therapies, Drug OR Chemotherapies OR Pharmacotherapy OR Pharmacotherapies OR Chemotherapy

#16 #14 or #15

#17 #4 or #7 or #10 or #13 or #16

#18 3L OR 3 line OR third line OR third-line OR Previously Treated OR Treated OR Later Treated OR pretreated OR placebo

#19 #3 and #17 and #18

**Supplementary Table 3**

Comparisons of the fit of consistency and inconsistency models using deviance information criteria (DIC), statistical heterogeneity of primary and secondary outcomes.

|  |  | **model** | | | **I^2^** |
| --- | --- | --- | --- | --- | --- |
|  |  | **inconsistency** | **consistency, random** | **consistency, fixed** |  |
| **overall** | **OS** | 16.92 | 16.89 | 17.15 | 34% |
|  | **PFS** | 16.72 | 16.73 | 16.75 | 32% |
|  | **DCR** | 27.68 | 27.46 | 28.61 | 26% |
|  | **≥3AEs** | 15.96 | 15.99 | 16.01 | 13% |
| **Age****≥65** | **OS** | 11.27 | 11.32 | 9.92 | 0% |
| **Age<65** | **OS** | 11.97 | 11.94 | 10.76 | 11% |
| **Male** | **OS** | 10.33 | 10.63 | 8.57 | 0% |
| **Female** | **OS** | 15.47 | 15.41 | 16.44 | 44% |
| **ECOG PS=0** | **OS** | 13.69 | 13.67 | 12.35 | 16% |
| **ECOG PS=1** | **OS** | 11.57 | 11.59 | 11.75 | 35% |
| **KRAS status: Mutated** | **OS** | 11.01 | 11.06 | 10.44 | 33% |
| **KRAS status: Wild-type** | **OS** | 9.27 | 9.33 | 8.30 | 6% |
| **Primary tumor site: Colon** | **OS** | 8.31 | 8.44 | 7.17 | 4% |
| **Primary tumor site: Rectum** | **OS** | 8.11 | 7.94 | 6.79 | 0% |
| **Time since diagnosis of first metastases: <18 months** | **OS** | 8.73 | 8.80 | 7.86 | 18% |
| **Time since diagnosis of first metastases: ≥18 months** | **OS** | 8.22 | 8.21 | 6.95 | 0% |

*OS, overall survival; PFS, progression-free survival; DCR, disease control rate; ≥3AEs, adverse events of grade 3 or higher;* *ECOG PS,* *Eastern Cooperative Oncology Group Performance Status.*

*.*
