# Supplementary material for: Comparison of the efficacy and safety of third-line treatments for metastatic colorectal cancer: a systematic review and network meta-analysis
Source: Front Oncol. 2023 Sep 21;13:1269203. doi: 10.3389/fonc.2023.1269203 (PMC10552753; doi:10.3389/fonc.2023.1269203)
Supplement: Supplementary file 2 [file DataSheet_2.pdf]

A

Treatment	/	/	Regorafenib	TAS-102	Fruquintinib	Regorafenib 80+	TAS-102 + Bevacizumab	Placebo
Overall	/	OS	0.33	0.44	0.50	0.76	**0.96**	0.00
	/	PFS	0.34	0.34	0.80	0.53	**0.99**	0.00
	/	DCR	0.52	0.33	**0.94**	NA	0.71	0.00
	/	≥3AEs	**0.96**	0.31	0.79	NA	0.44	0.00
Age	Age≥65	OS	0.41	0.73	0.24	NA	**1.00**	0.13
	Age<65	OS	0.53	0.28	0.79	NA	**0.90**	0.00
Gender	Male	OS	0.34	0.43	0.77	NA	**0.96**	0.00
	Female	OS	0.64	0.52	0.29	NA	**0.99**	0.06
ECOG	PS=0	OS	0.43	0.38	0.81	NA	**0.88**	0.00
	PS=1	OS	0.51	0.49	0.53	NA	**0.97**	0.00
KRAS status	Mutated	OS	0.53	**0.73**	0.71	NA	NA	0.04
	Wild type	OS	0.61	0.55	**0.84**	NA	NA	0.00
Primary tumor site	Colon	OS	0.67	**0.70**	0.61	NA	NA	0.02
	Rectum	OS	0.23	0.76	**0.87**	NA	NA	0.14
Time from first diagnosis of metastatic disease to randomisation	<18 months	OS	0.64	0.34	NA	NA	**0.98**	0.04
	≥18 months	OS	0.40	0.60	NA	NA	**0.99**	0.00

B

Treatment	/	/	Regorafenib	TAS-102	Fruquintinib	Regorafenib 80+	TAS-102 + Bevacizumab	Placebo
Overall	/	OS	5	4	3	2	**1**	6
	/	PFS	4	5	2	3	**1**	6
	/	DCR	3	4	**1**	NA	2	5
	/	≥3AEs	**1**	4	2	NA	3	5
Age	Age≥65	OS	3	2	4	NA	**1**	5
	Age<65	OS	3	4	2	NA	**1**	5
Gender	Male	OS	4	3	2	NA	**1**	5
	Female	OS	2	3	4	NA	**1**	5
ECOG	PS=0	OS	3	4	2	NA	**1**	5
	PS=1	OS	3	4	2	NA	**1**	5
KRAS status	Mutated	OS	3	**1**	2	NA	NA	4
	Wild type	OS	2	3	**1**	NA	NA	4
Primary tumor site	Colon	OS	2	**1**	3	NA	NA	4
	Rectum	OS	3	2	**1**	NA	NA	4
Time from first diagnosis of metastatic disease to randomisation	<18 months	OS	2	3	NA	NA	**1**	4
	≥18 months	OS	3	2	NA	NA	**1**	4

Supplementary Figure 1

Bayesian ranking profiles of comparable treatments for efficacy and safety in mCRC patients.

(A) The surface value under the cumulative ranking curve (SUCRA) in each cell represents the probability of each treatment being ranked from first (high value) to last (low value) in terms of overall survival (OS), OS for subgroups, progression-free survival (PFS), disease control rate (DCR), and adverse events of grade 3 or higher (≥3 AEs), with the probability of the value ranking first indicated in bold. (B) The number in each cell indicates the probability of each treatment being ranked from first to last on OS, PFS, DCR, ⩾3AEs, and OS subgroups according to the value of the surface under the cumulative ranking curve (SUCRA).

Abbreviations: OS, overall survival; PFS, progression-free survival; DCR, disease control rate; ≥3AEs, adverse events of grade 3 or higher; ECOG PS, Eastern Cooperative Oncology Group Performance Status; TAS-102, trifluridine/tipiracil; Regorafenib 80+, regorafenib dose-escalation regimen; BSC, best supportive.

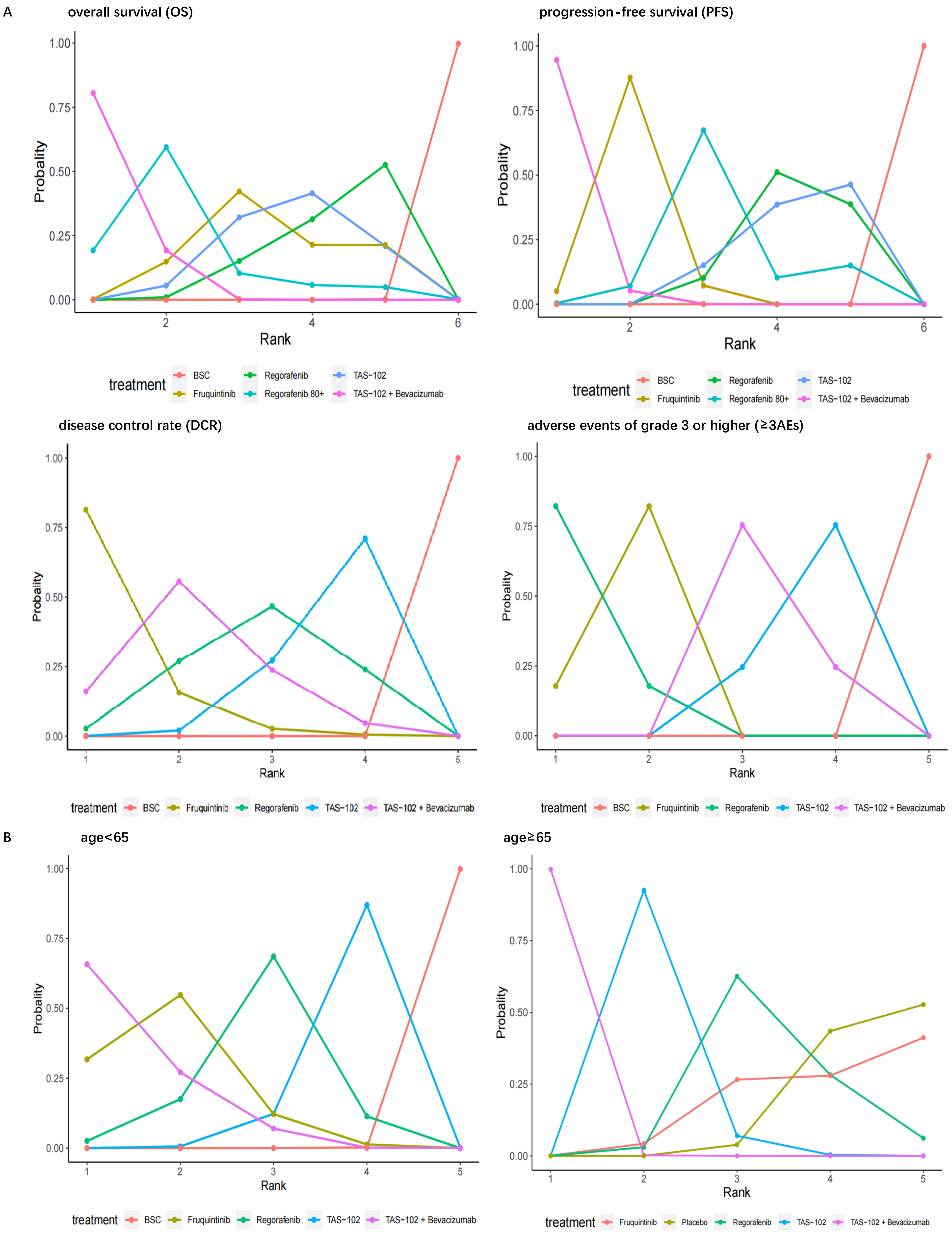
A
overall survival (OS)
Probality
Rank
treatment BSC Fruquintinib Regorafenib Regorafenib 80+ TAS−102 TAS−102 + Bevacizumab
progression-free survival (PFS)
Probality
Rank
treatment BSC Fruquintinib Regorafenib Regorafenib 80+ TAS−102 TAS−102 + Bevacizumab
disease control rate (DCR)
Probality
Rank
treatment BSC Fruquintinib Regorafenib TAS−102 TAS−102 + Bevacizumab
adverse events of grade 3 or higher (≥3AEs)
Probality
Rank
treatment BSC Fruquintinib Regorafenib TAS−102 TAS−102 + Bevacizumab
B
age<65
Probality
Rank
treatment BSC Fruquintinib Regorafenib TAS−102 TAS−102 + Bevacizumab
age≥65
Probality
Rank
treatment Fruquintinib Placebo Regorafenib TAS−102 TAS−102 + Bevacizumab

male

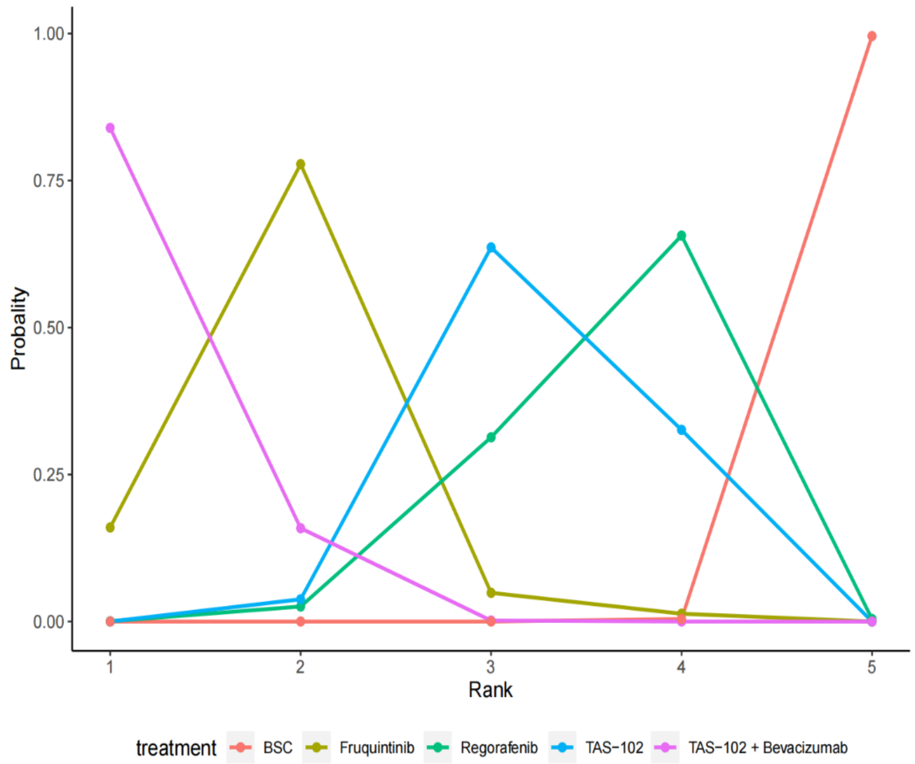

female

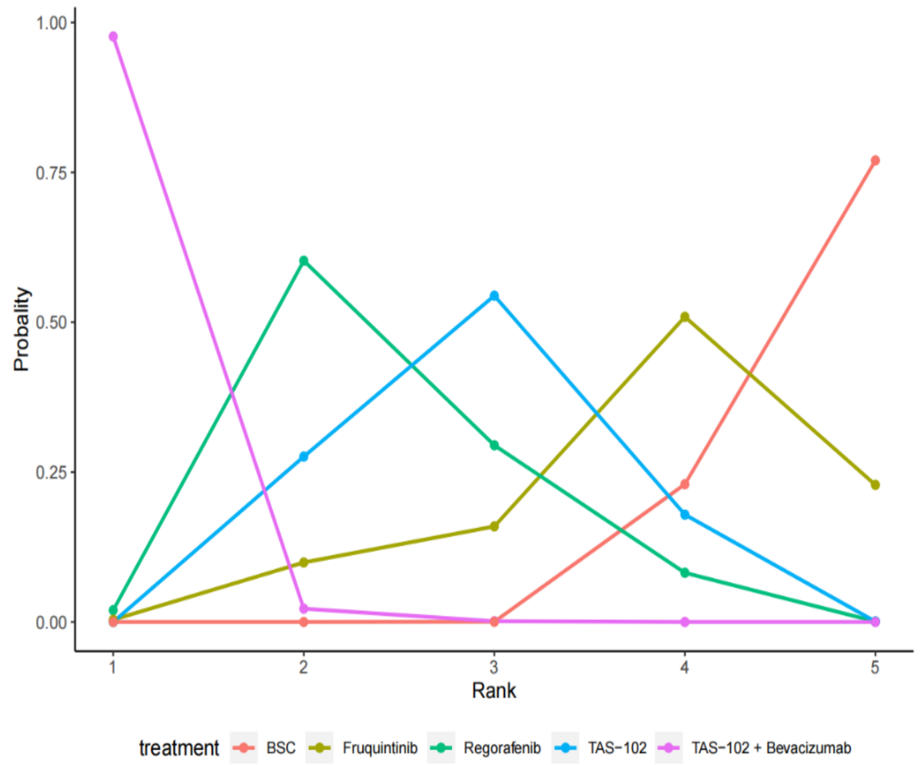

C

ECOG PS=0

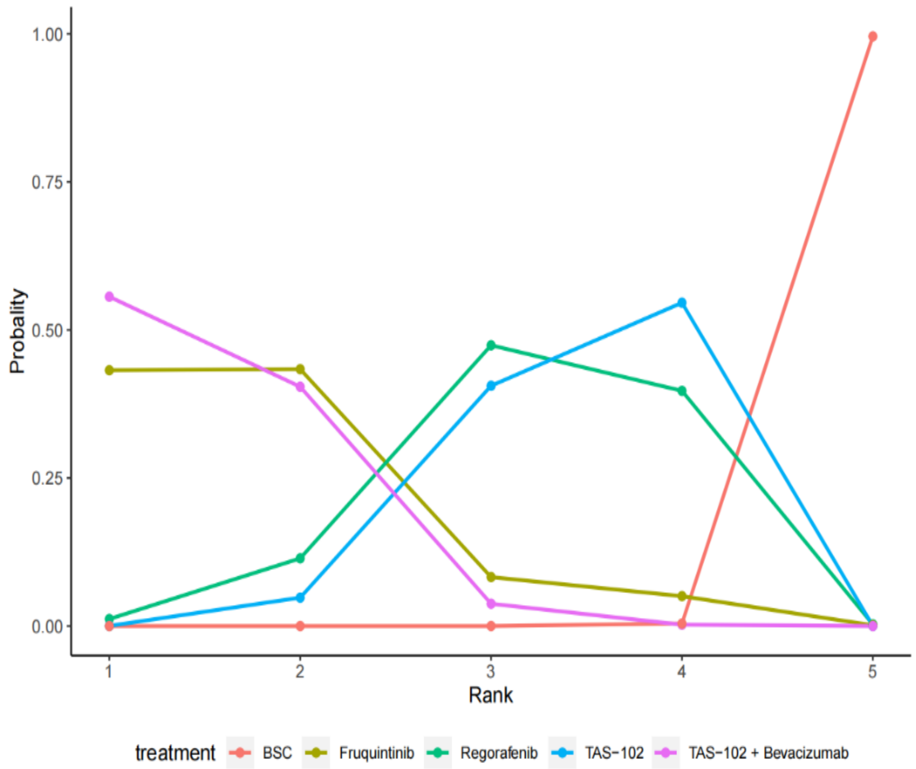

ECOG PS=1

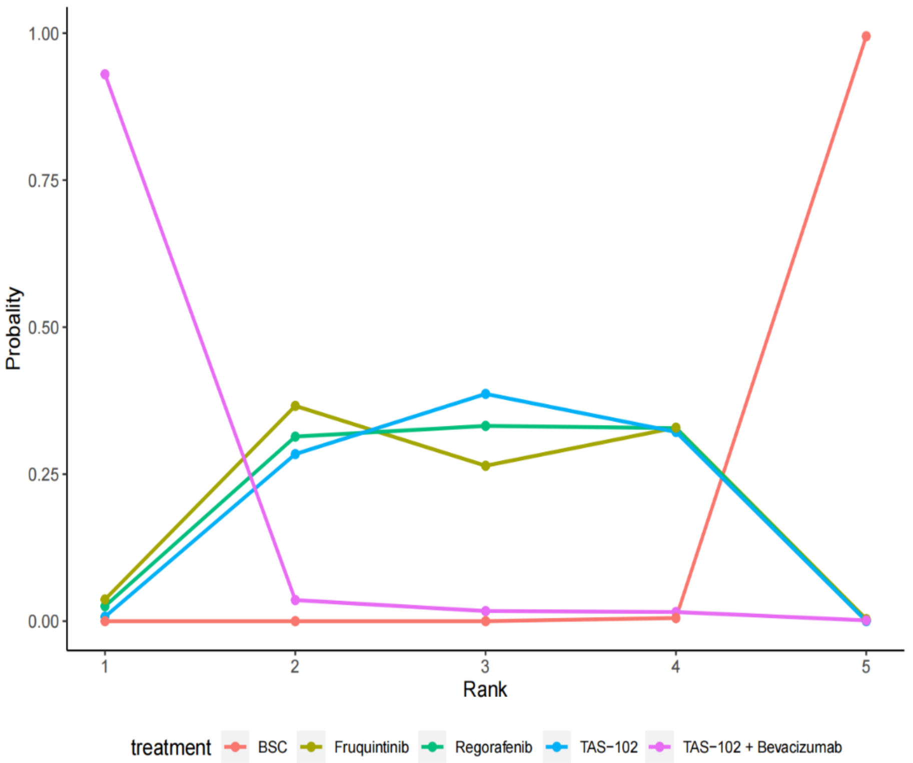

KRAS mutant

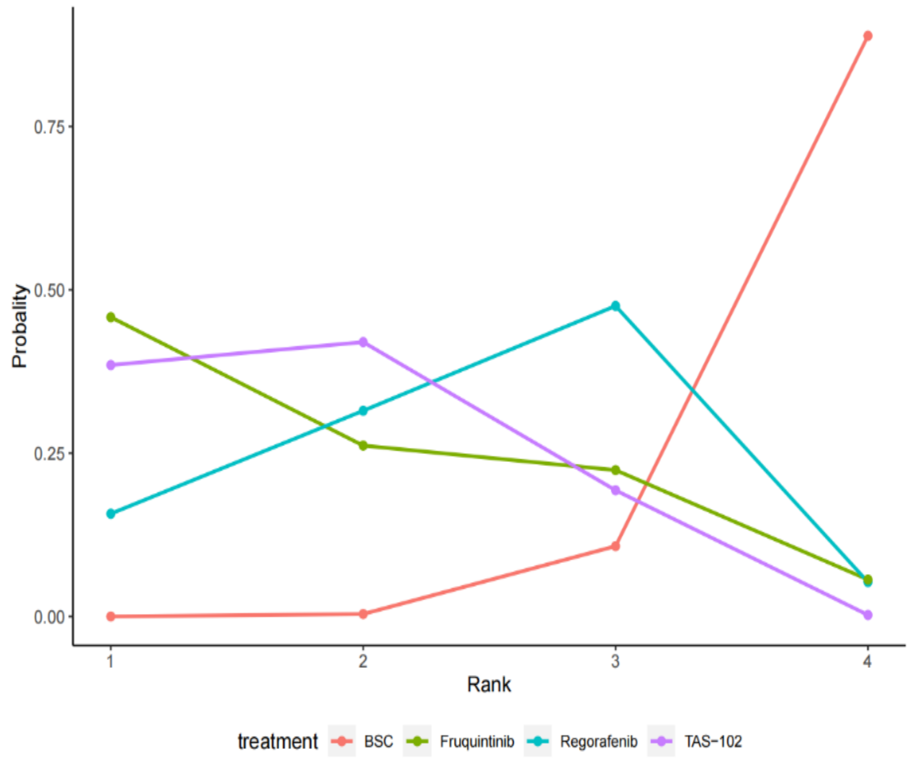

KRAS wild-type

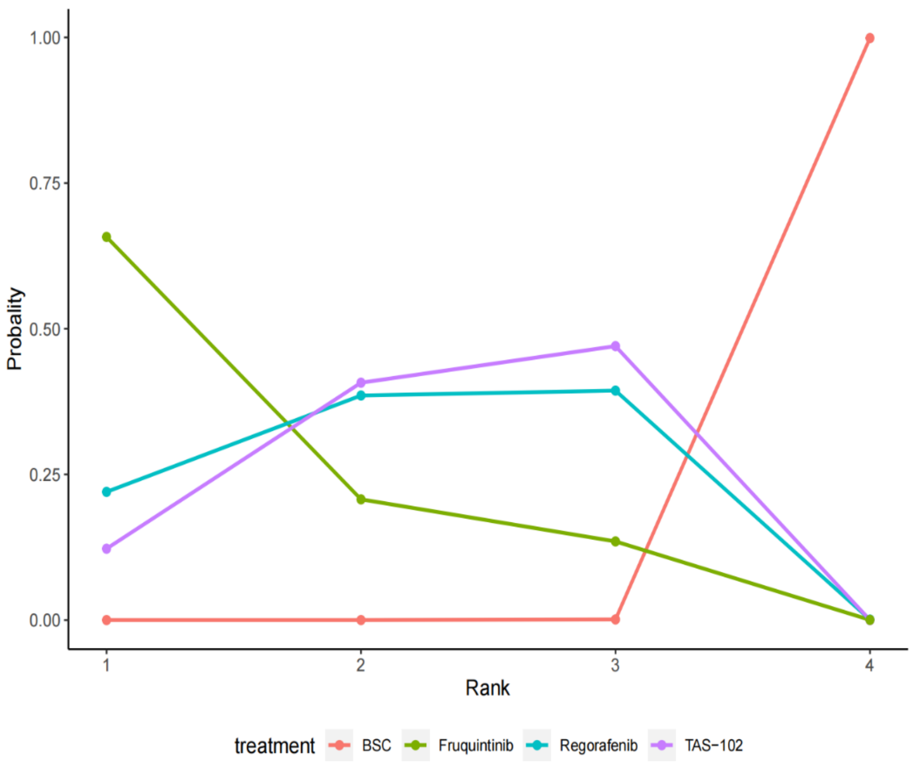

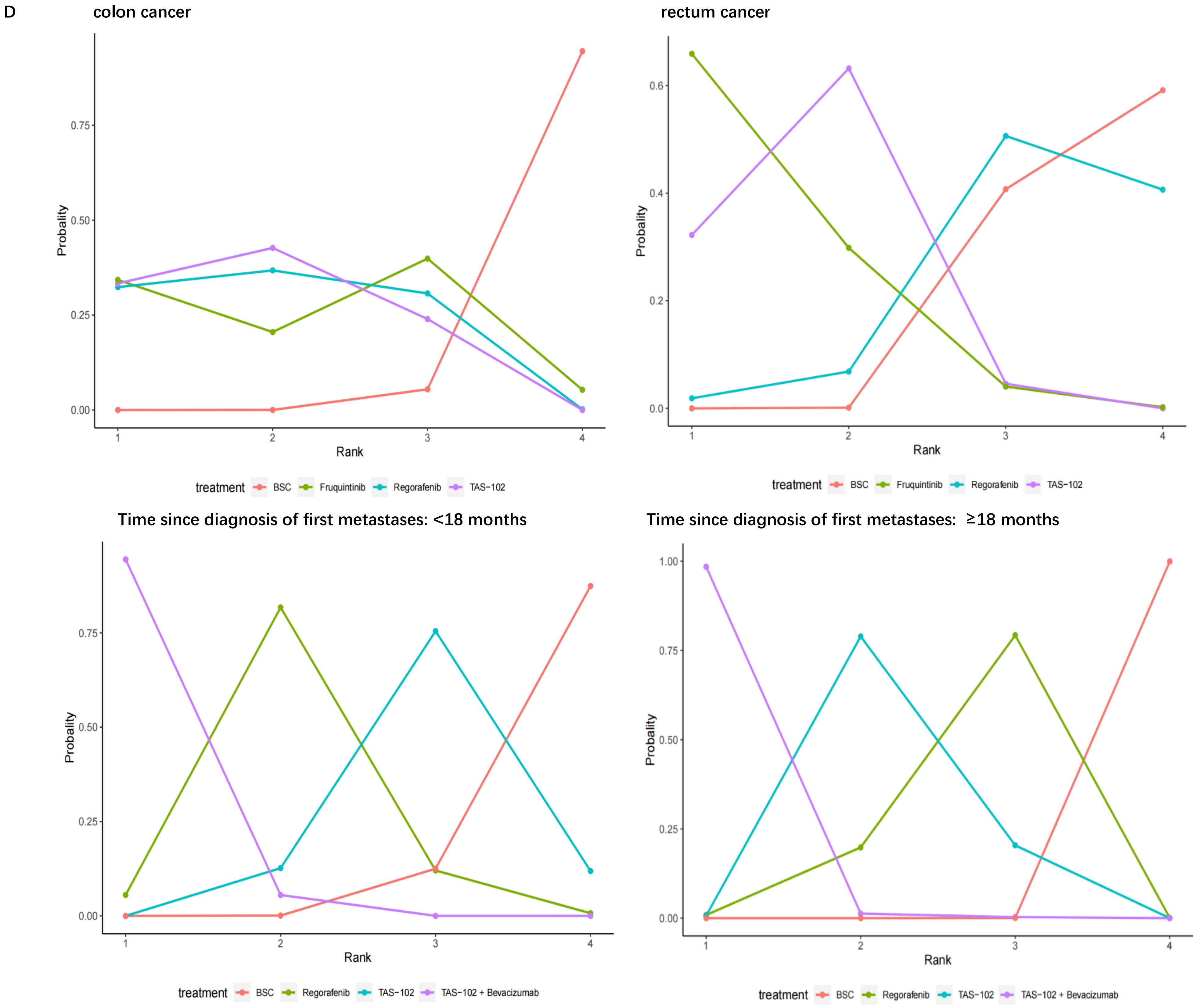

Supplementary Figure 2

Bayesian ranking profiles of comparable treatments for mCRC patients in terms of efficacy and safety.

Ranking curves indicate the probability of ranking each comparable treatment from first to last. (A) Overall survival (OS), progression-free survival (PFS), disease control rate (DCR), adverse events of grade 3 or higher (≥3 AEs). (B) OS for age≥65, age<65 subgroup, male, female subgroup. (C) OS for ECOG PS=0, ECOG PS=1, KRAS wild-type, KRAS mutant subgroup. (D) OS for colon cancer, rectum cancer, diagnosis of metastatic disease <18 months, ≥ 18 months subgroup.

Abbreviations: ECOG PS, Eastern Cooperative Oncology Group Performance Status.

	NO.of studies	Sample size	Regorafenib	TAS-102	Fruquintinib	Regorafenib 80+	TAS-102 + Bevacizumab	BSC
**Fatigue**	9	3456	1.82	1.33	0.09	0.20	0.17	0.90
**Hand-foot skin reaction**	4	1496	7.69	NA	2.01	0.53	NA	0.07
**Diarrhoea**	9	3456	1.13	0.90	0.23	0.03	0.17	0.14
**Hypertension**	6	2394	2.51	0.17	2.46	0.17	0.58	0.29
**Decreased appetite/Anorexia**	8	3363	0.56	0.86	0.09	0.03	0.06	0.65
**Vomiting**	7	2836	0.14	0.78	NA	0.00	0.14	0.04
**Oral mucositis**	2	876	1.83	NA	NA	0.11	NA	0.00
**Rash or desquamation**	1	760	3.82	NA	NA	NA	NA	NA
**Hypophoshpataemia**	2	964	2.90	NA	NA	NA	NA	0.10
**Anaemia**	8	3040	0.56	6.51	NA	0.03	0.56	0.63
**Thrombocytopenia**	9	3456	0.52	1.24	0.20	0.00	NA	0.12
**Bilirubin level elevated**	6	2702	0.89	2.37	0.15	0.07	NA	1.70
**Proteinuria**	3	1380	0.65	NA	0.65	NA	NA	0.14
**Neutropenia**	6	2164	0.14	20.52	NA	NA	6.33	0.00
**Leucopenia**	4	1579	0.19	12.73	NA	NA	NA	0.00
**Lymphopenia**	3	691	0.00	7.24	NA	0.58	NA	0.72
**Thrombocytopenia**	9	3456	0.52	1.24	0.20	0.00	0.23	0.12
**Increase alkaline phosphatase level**	4	1526	0.13	3.47	NA	0.20	NA	2.23

Supplementary Figure 3

A frequency toxicity profile in relation to the incidence (%) of each adverse events of grade 3 or higher (≥3 AEs) based on the population of each treatment in NMA we included.

Abbreviations: TAS-102, trifluridine/tipiracil; Regorafenib 80+, regorafenib dose-escalation regimen; BSC, best supportive.

A

Age＜65

Age≥65

**Regorafenib**	1.17 (0.90, 1.52)	0.83 (0.60, 1.16)	0.76 (0.51, 1.12)	1.49 (1.22, 1.82)
1.43 (0.98, 2.07)	**TAS-102**	0.71 (0.52, 0.97)	0.65 (0.48, 0.87)	1.27 (1.08, 1.50)
0.86 (0.46, 1.61)	0.60 (0.34, 1.08)	**Fruquintinib**	0.91 (0.59, 1.40)	1.78 (1.37, 2.33)
2.44 (1.48, 4.02)	1.71 (1.23, 2.38)	2.85 (1.45, 5.54)	**TAS-102 + Bevacizumab**	1.97 (1.40, 2.77)
0.81 (0.60, 1.11)	0.57 (0.46, 0.70)	0.95 (0.55, 1.63)	0.33 (0.23, 0.49)	**BSC**

OS

B

Female

Male

**Regorafenib**	1.09 (0.77, 1.52)	1.30 (0.80, 2.11)	0.63 (0.41, 0.98)	1.52 (1.17, 1.97)
1.06 (0.81, 1.40)	**TAS-102**	1.20 (0.76, 1.9)	0.58 (0.44, 0.77)	1.40 (1.13, 1.72)
1.42 (0.98, 2.06)	1.34 (0.96, 1.87)	**Fruquintinib**	0.48 (0.28, 0.83)	1.17 (0.78, 1.75)
1.77 (1.20, 2.61)	1.66 (1.27, 2.19)	1.25 (0.81, 1.92)	**TAS-102 + Bevacizumab**	2.41 (1.69, 3.43)
0.74 (0.59, 0.93)	0.70 (0.59, 0.82)	0.52 (0.39, 0.70)	0.42 (0.30, 0.58)	**BSC**

OS

C

ECOG PS=1

ECOG PS=0

**Regorafenib**	1.00 (0.76, 1.33)	0.99 (0.7, 1.41)	0.56 (0.29, 1.09)	1.45 (1.16, 1.8)
0.97 (0.7, 1.34)	**TAS-102**	0.98 (0.71, 1.37)	0.56 (0.31, 1.01)	1.44 (1.20, 1.72)
1.39 (0.82, 2.37)	1.44 (0.87, 2.40)	**Fruquintinib**	0.57 (0.29, 1.12)	1.46 (1.11, 1.92)
1.46 (0.94, 2.29)	1.51 (1.12, 2.05)	1.05 (0.58, 1.89)	**TAS-102 + Bevacizumab**	2.57 (1.39, 4.77)
0.69 (0.53, 0.90)	0.71 (0.59, 0.87)	0.49 (0.31, 0.79)	0.47 (0.33, 0.68)	**BSC**

OS

D

Wild-type

Mutant

**Regorafenib**	0.92 (0.68, 1.24)	0.91 (0.60, 1.39)	0.54 (0.37, 0.79)	1.21 (0.96, 1.53)
1.09 (0.81, 1.47)	**TAS-102**	0.99 (0.67, 1.48)	0.59 (0.46, 0.75)	1.32 (1.09, 1.60)
1.10 (0.72, 1.67)	1.01 (0.68, 1.50)	**Fruquintinib**	0.60 (0.26, 1.37)	1.79 (1.28, 2.5)
1.85 (1.26, 2.72)	1.70 (1.34, 2.16)	1.69 (1.06, 2.69)	**TAS-102 + Bevacizumab**	2.25 (1.65, 3.04)
0.83 (0.65, 1.04)	0.76 (0.63, 0.92)	0.75 (0.53, 1.07)	0.45 (0.33, 0.61)	**BSC**

KRAS status (OS)

E

Rectum (upper triangle) / Colon (lower triangle)

**Regorafenib**	0.69 (0.43, 1.09)	0.62 (0.36, 1.10)	1.05 (0.70, 1.59)
1.01 (0.76, 1.34)	**TAS-102**	0.91 (0.59, 1.40)	1.53 (1.24, 1.90)
0.97 (0.62, 1.54)	0.96 (0.63, 1.47)	**Fruquintinib**	1.68 (1.16, 2.44)
0.71 (0.56, 0.89)	0.70 (0.59, 0.82)	0.72 (0.49, 1.07)	**BSC**

Primary sites (OS)

F

≥18 months (upper triangle) / <18 months (lower triangle)

**Regorafenib**	0.90 (0.69, 1.16)	0.63 (0.43, 0.93)	1.38 (1.13, 1.68)
0.79 (0.53, 1.18)	**TAS-102**	0.71 (0.53, 0.94)	1.54 (1.30, 1.81)
1.53 (0.91, 2.59)	1.94 (1.39, 2.70)	**TAS-102 + Bevacizumab**	2.18 (1.56, 3.03)
0.68 (0.49, 0.93)	0.86 (0.66, 1.11)	0.44 (0.29, 0.67)	**BSC**

Time since diagnosis of first metastases (OS)

Supplementary Figure 4

Network meta-analysis of the third-line treatments for mCRC.

(A) Pooled Hazard Ratio (HR) (95% CrI (credible interval)) for OS of age ≥ 65 and < 65. (B) Pooled HR (95 CrI) for OS of female and male. (C) Pooled HR (95 CrI) for OS of Eastern Cooperative Oncology Group Performance Status = 0/1 (ECOG PS = 0, ECOG PS = 1). (D) Pooled HR (95 CrI) for OS of KRAS status (KRAS wild-type, KRAS mutant). (E) Pooled HR (95 CrI) for OS of primary sites (Colon, Rectum). (F) Pooled HR (95 CrI) for OS of time since diagnosis of first metastases (<18 months, ≥ 18 months). Significant results are highlighted in red.

Abbreviations: mCRC, metastatic colorectal cancer; TAS-102, trifluridine/tipiracil; BSC, best supportive care.

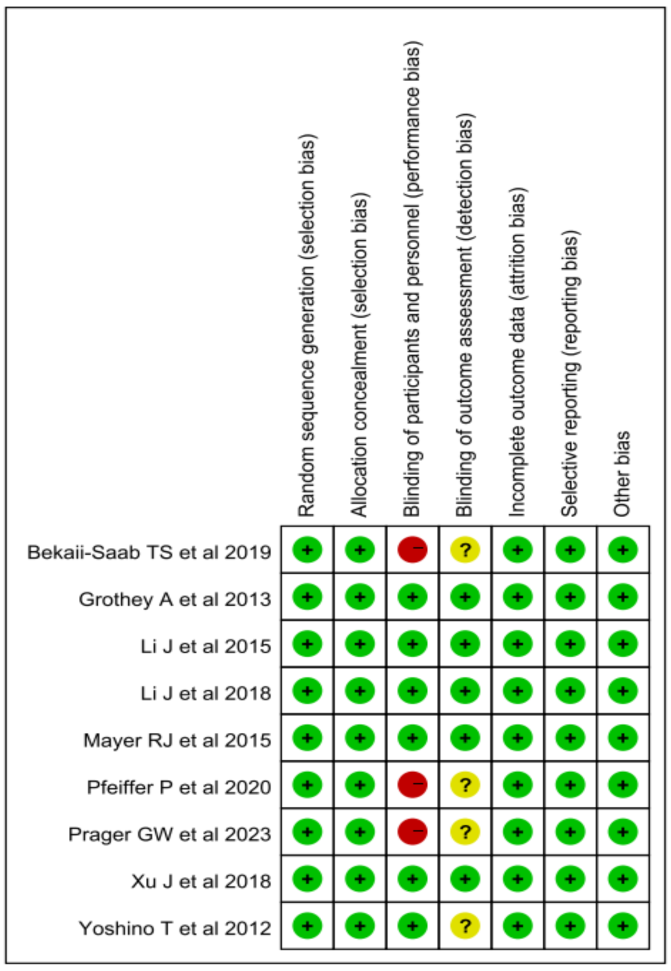

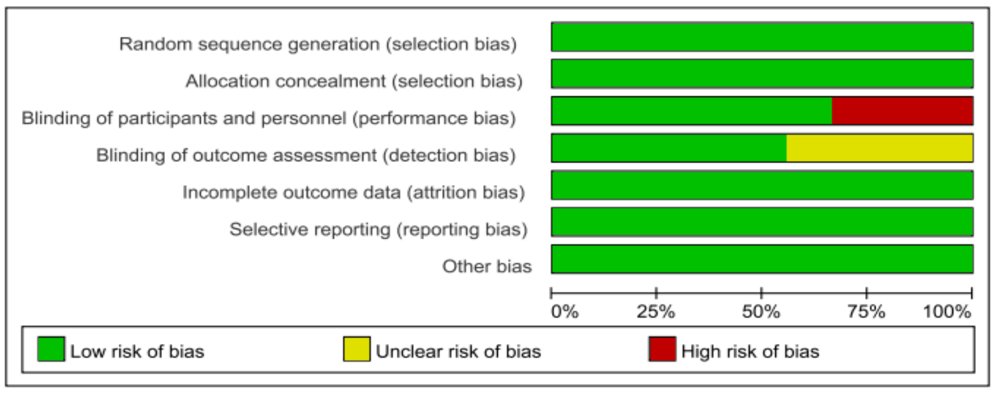

+: **low risk;** ?: **Unclear risk; - : high risk.**

Supplementary Figure 5

Summary of results from the bias risk assessment of RCTs using the Cochrane risk of bias tool.

OS

A. Brooks-Gelman-Rubin diagnostic

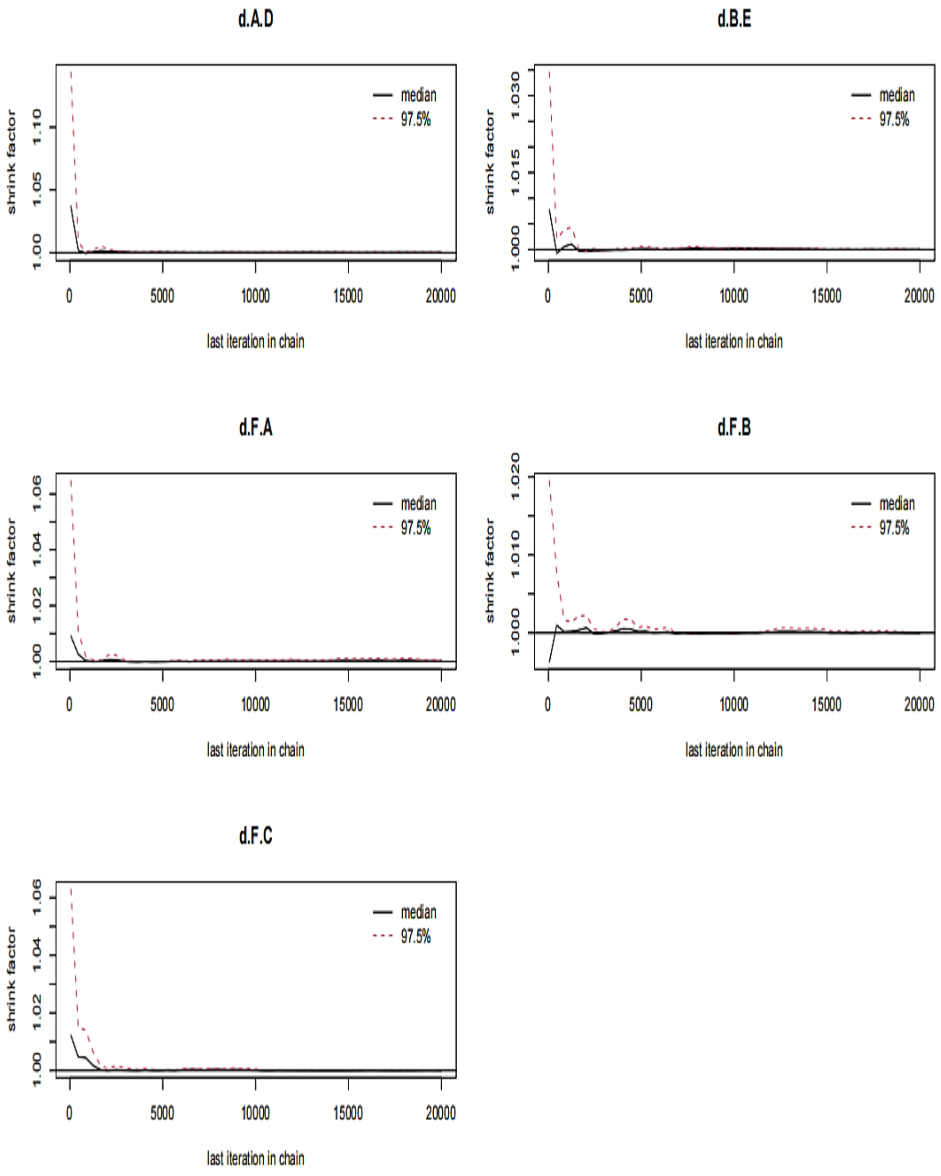

B. Trace plot

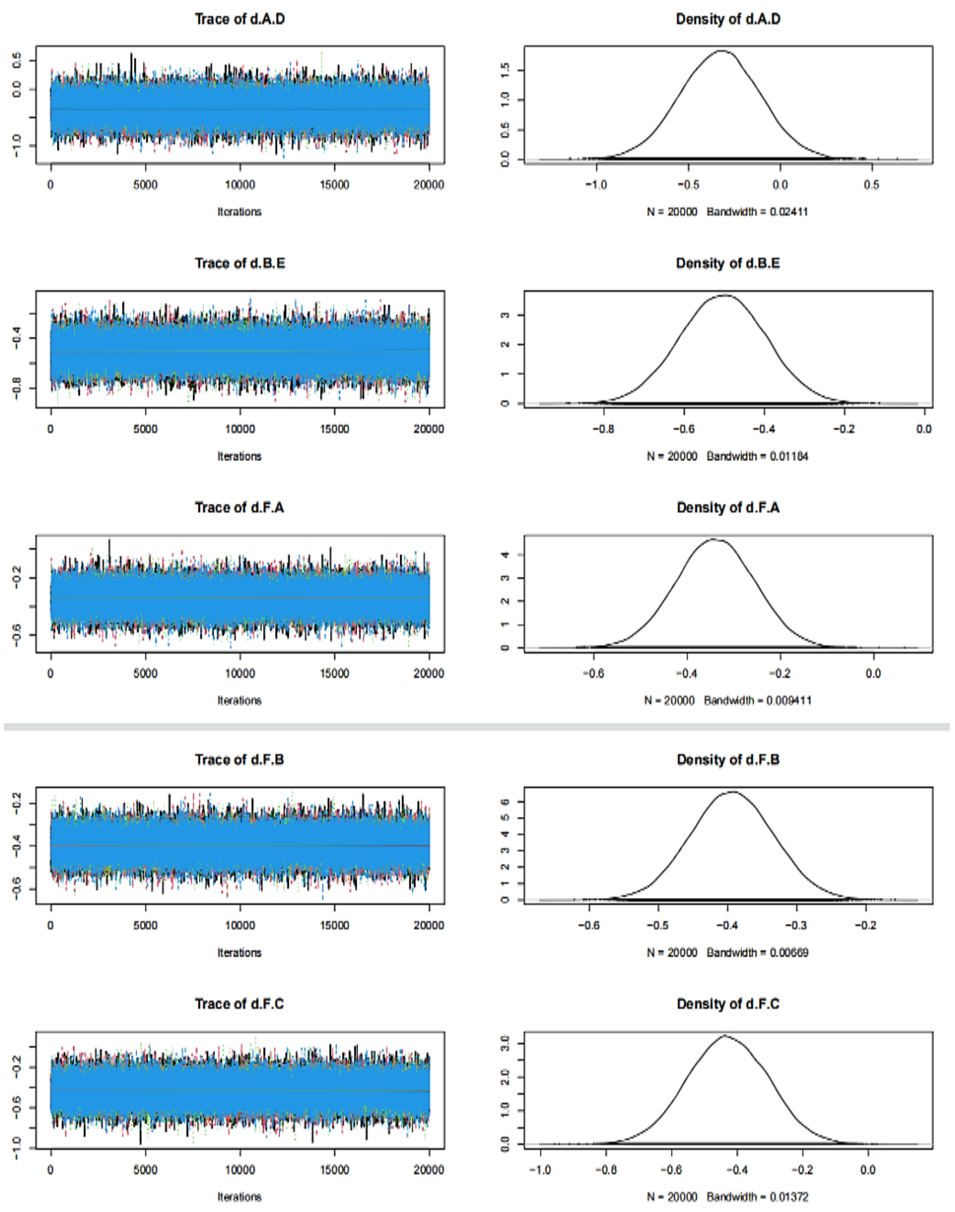

PFS

C. Brooks-Gelman-Rubin diagnostic

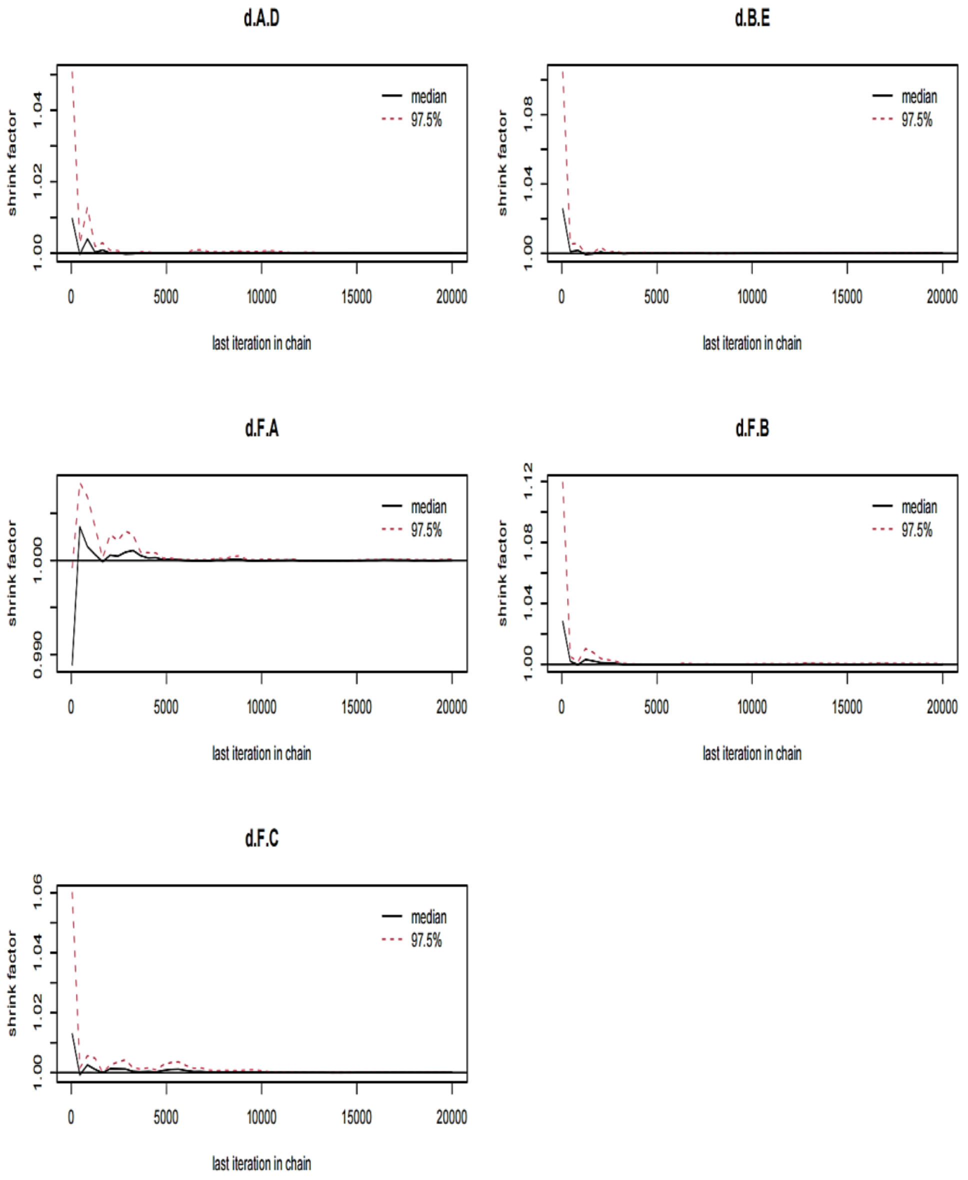

D. Trace plot

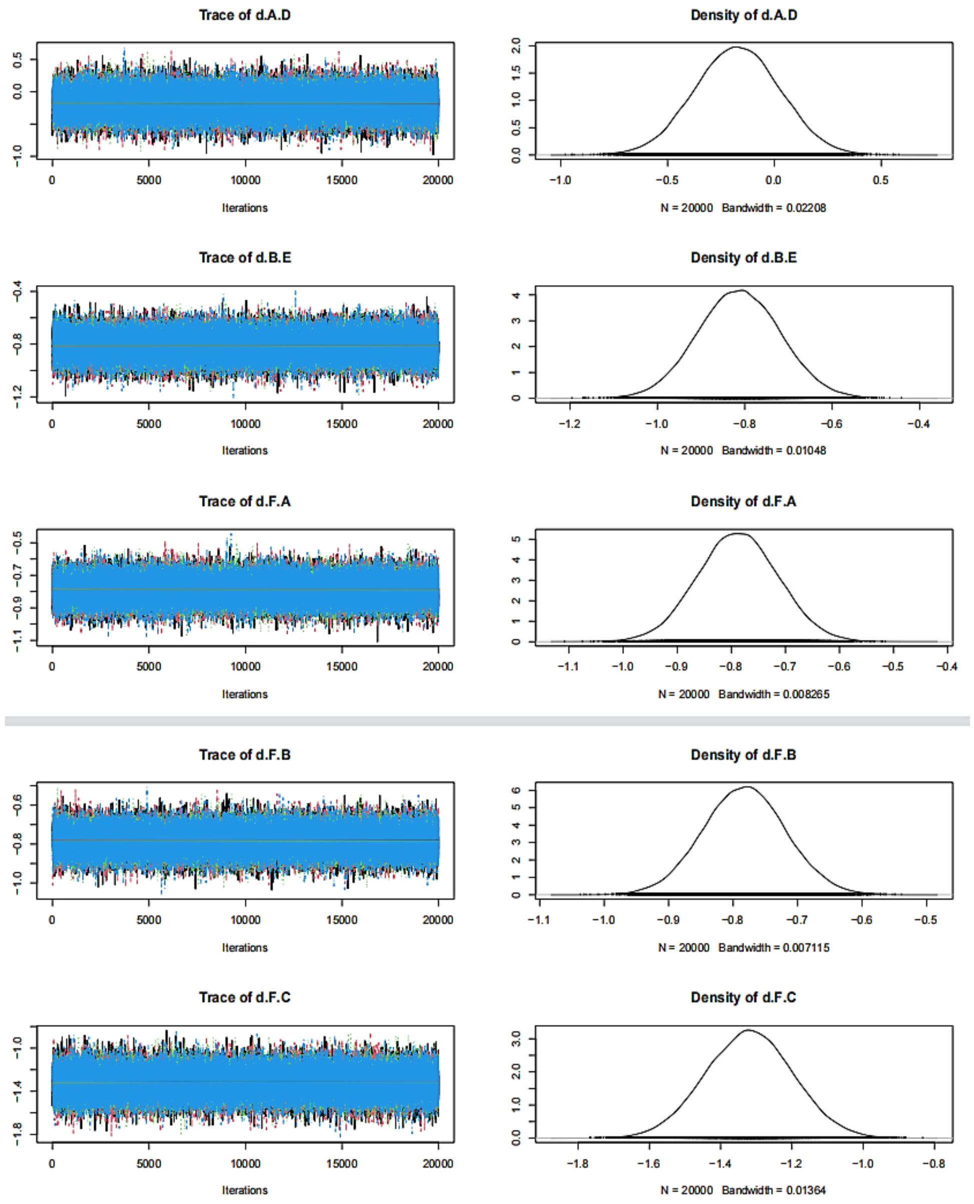

DCR

E. Brooks-Gelman-Rubin diagnostic

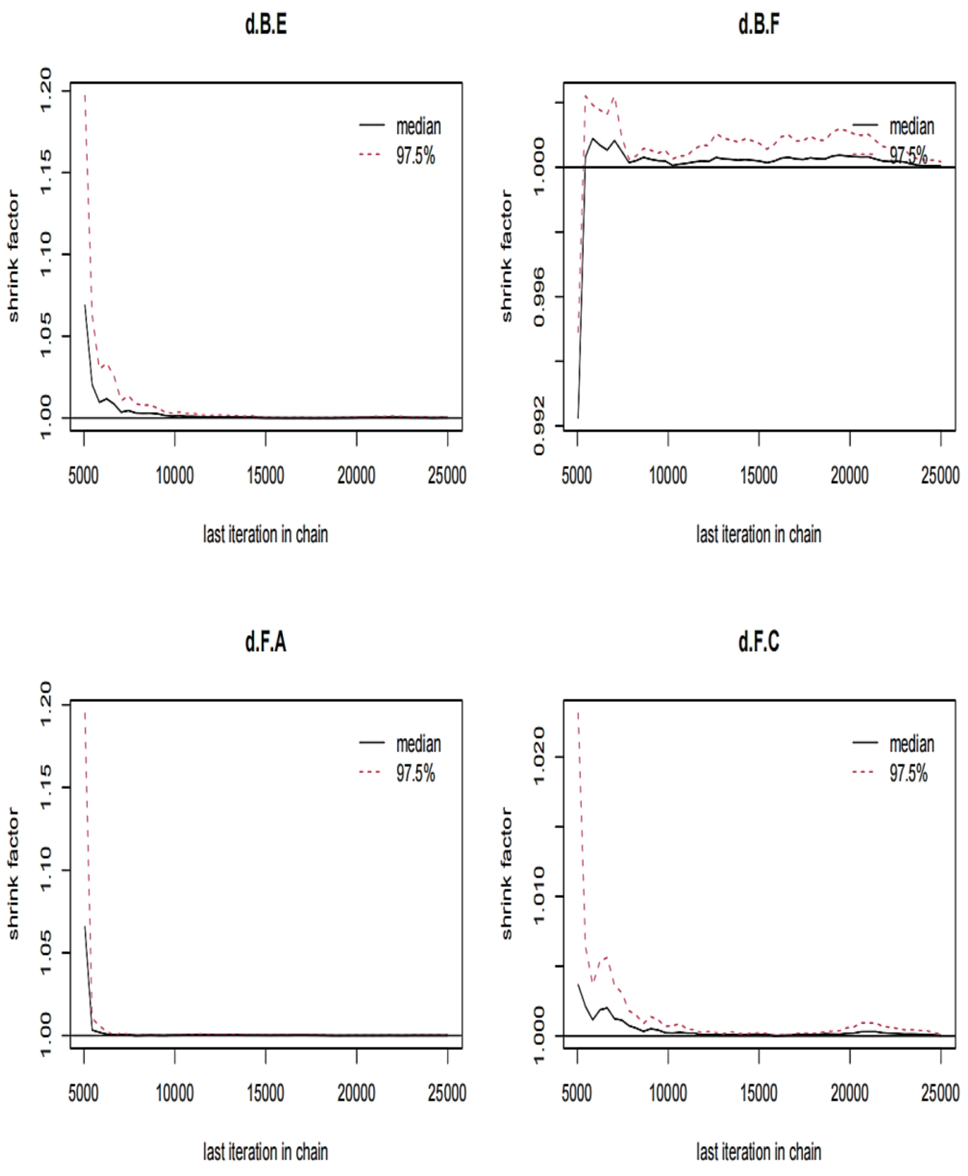

F. Trace plot

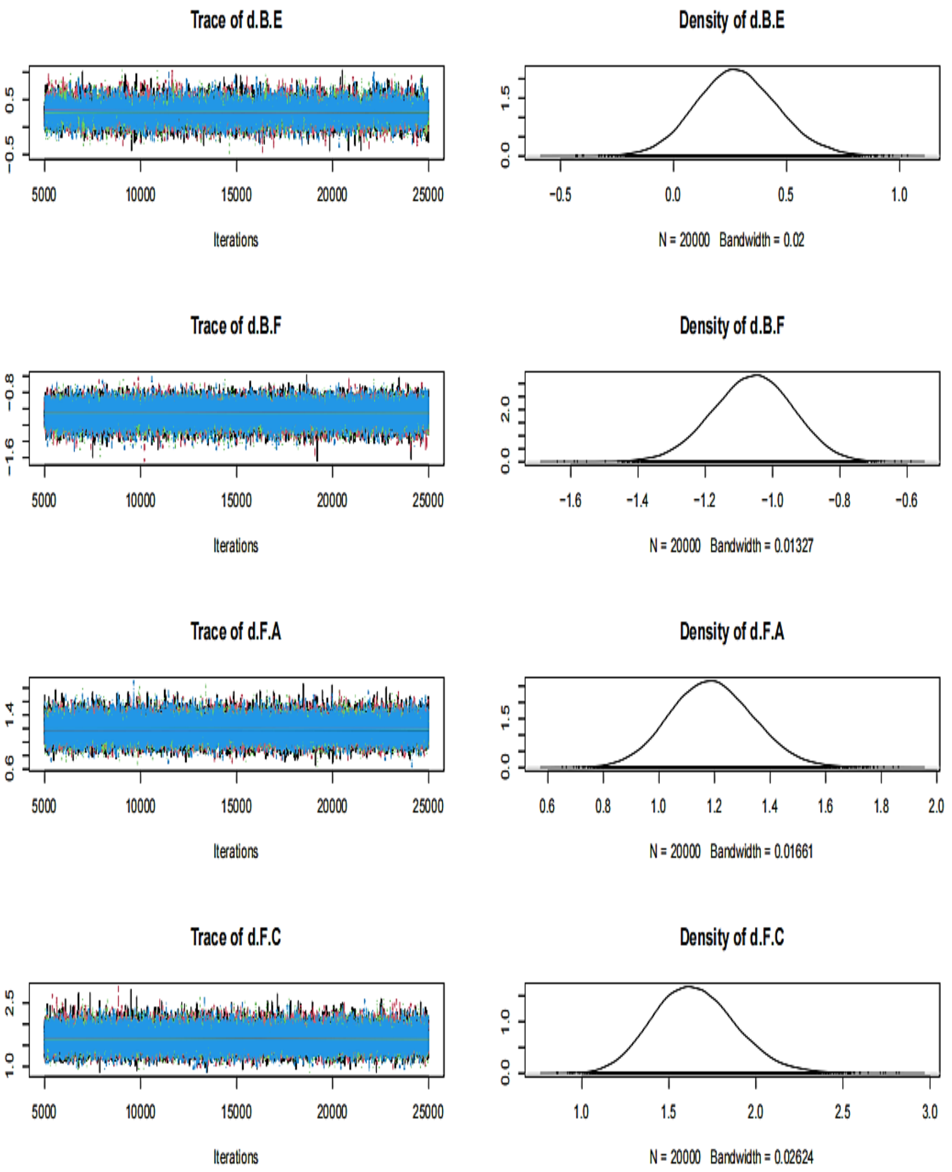

≥3AEs

G. Brooks-Gelman-Rubin diagnostic

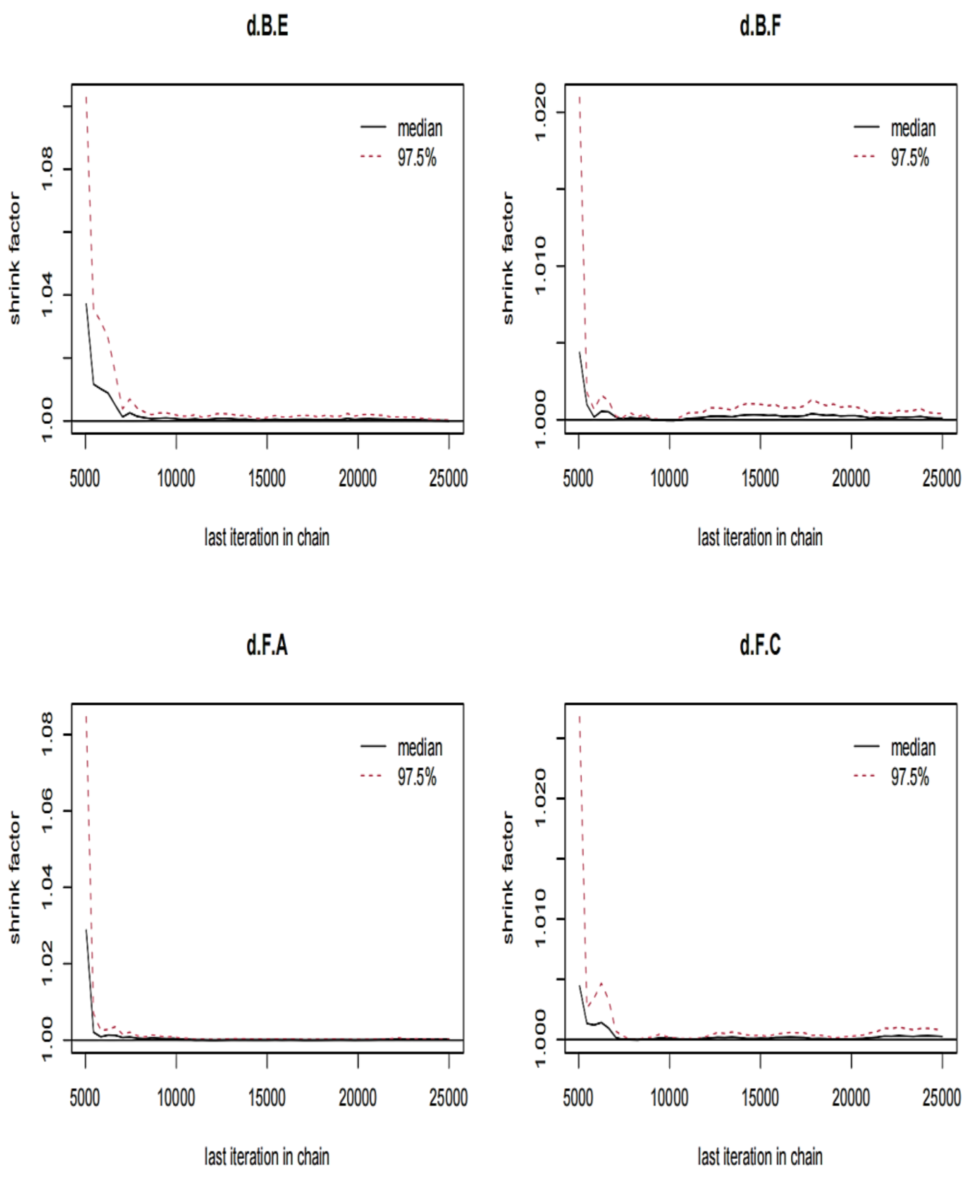

H. Trace plot

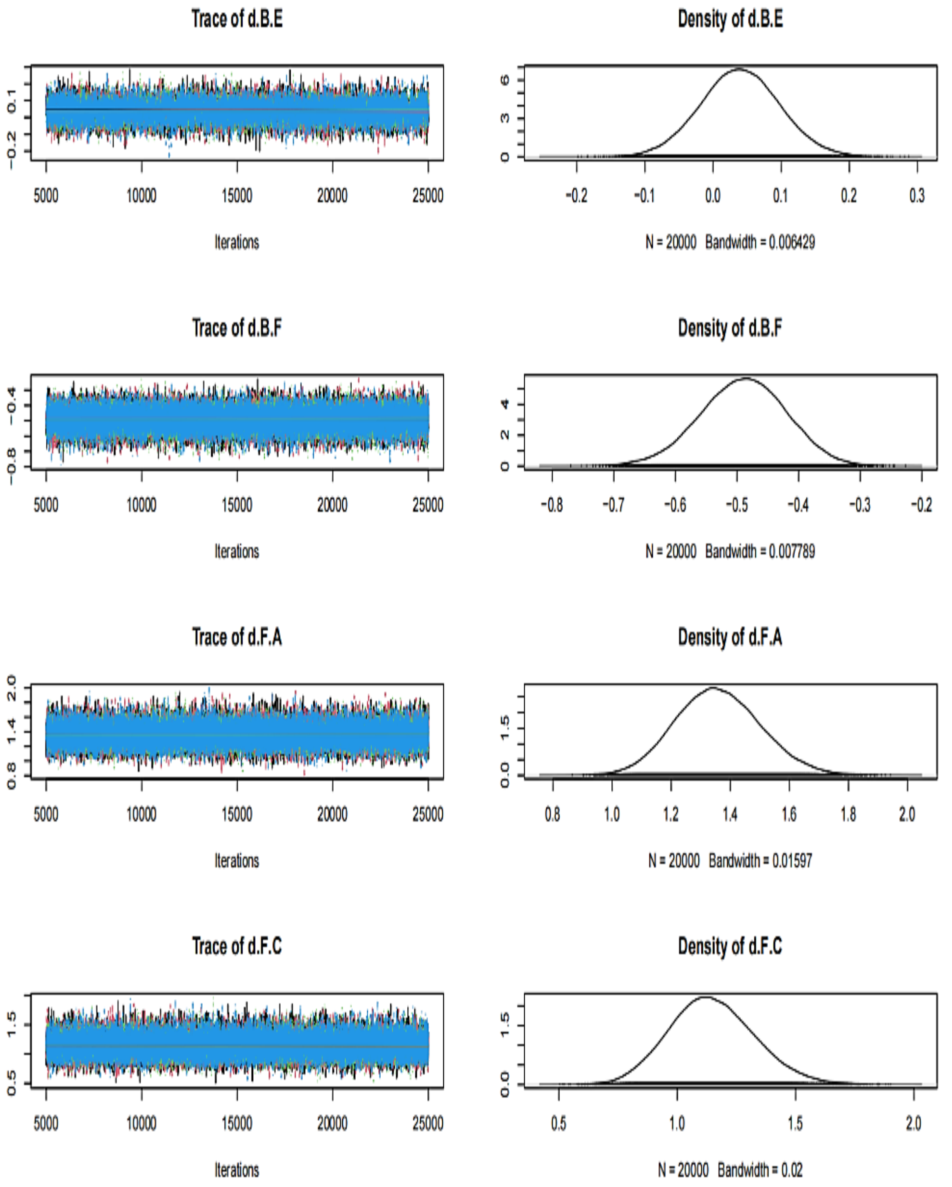

KRAS status: mutant

I. Brooks-Gelman-Rubin diagnostic

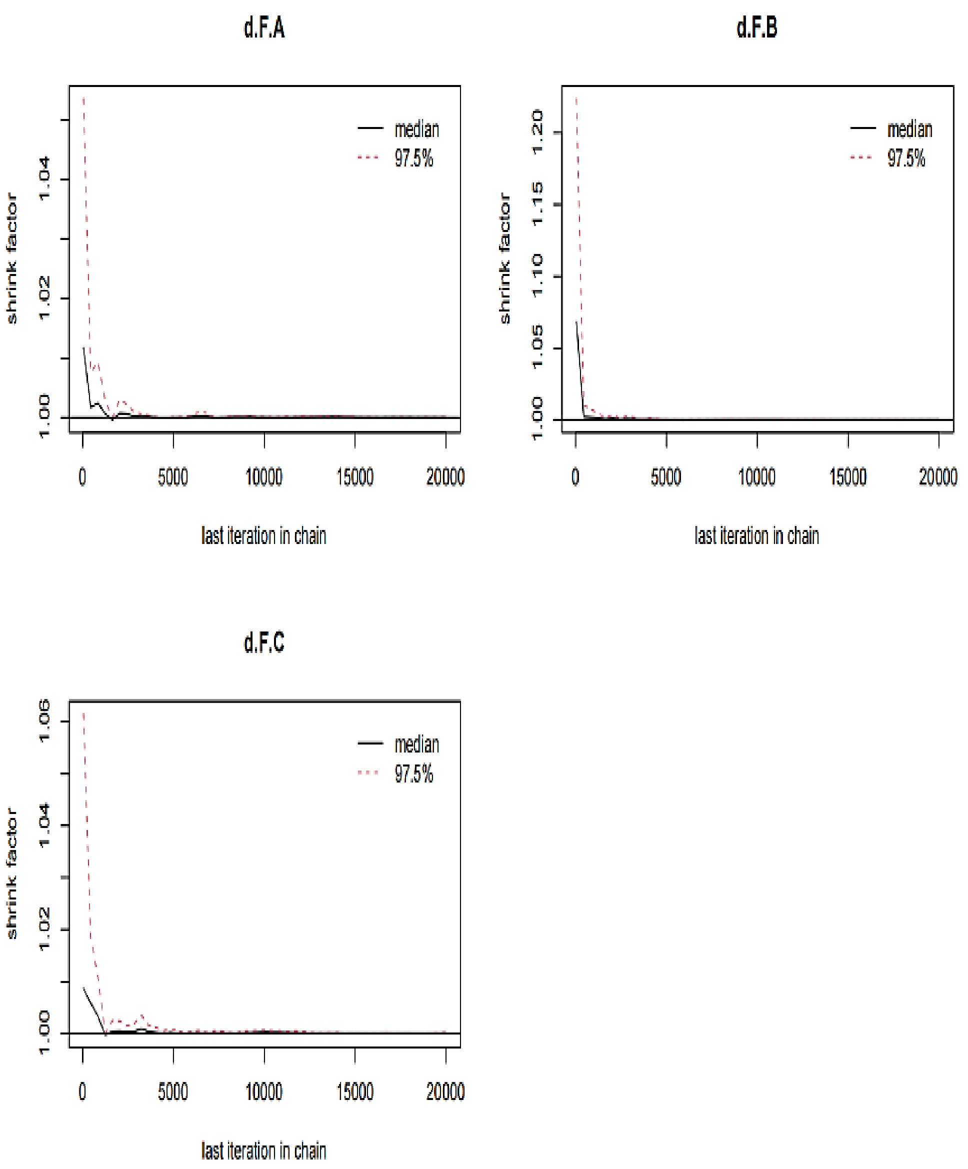

J. Trace plot

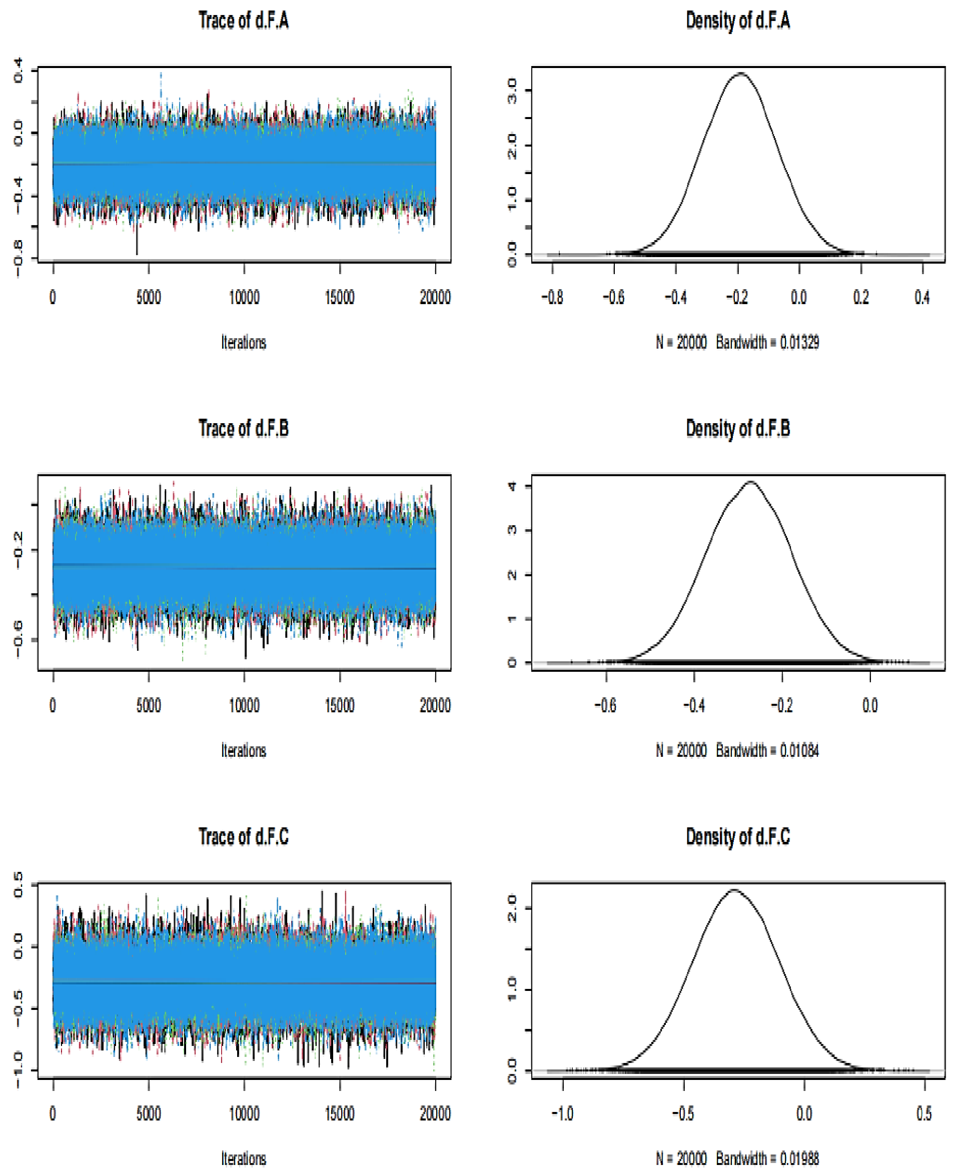

KRAS status: wild-type

K. Brooks-Gelman-Rubin diagnostic

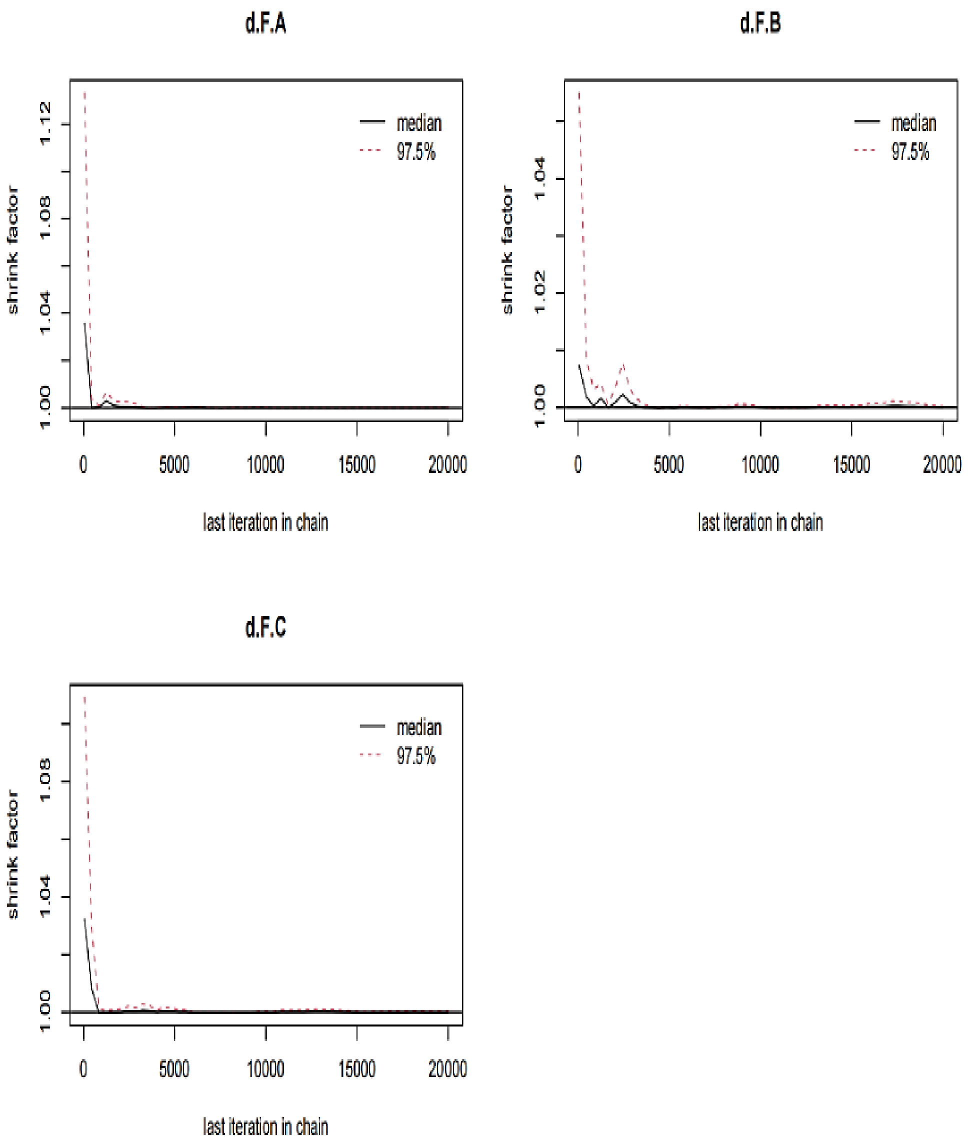

L. Trace plot

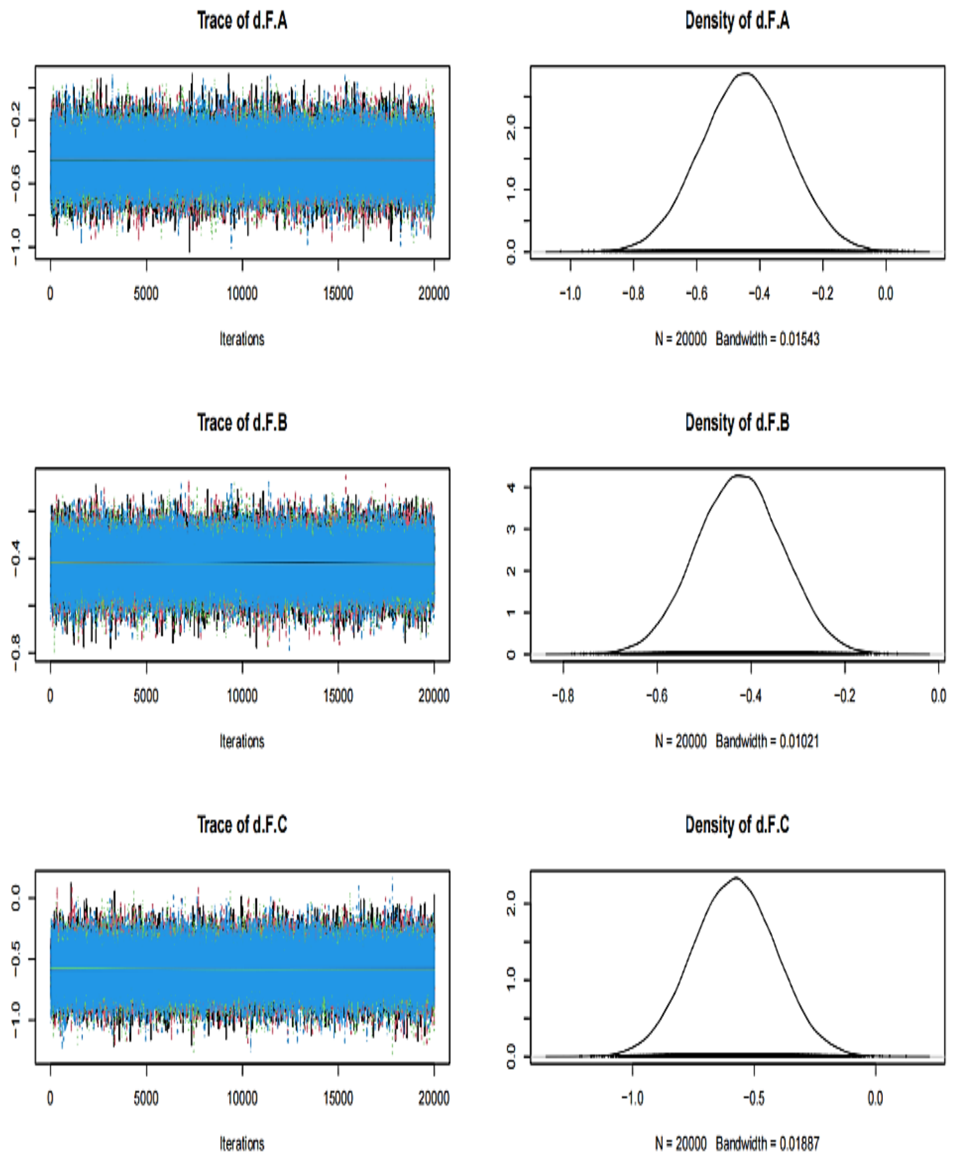

Supplementary Figure 6

Convergence of the four chains established by inspection of the Brooks-Gelman-Rubin diagnostic and the density trace plot. Overall survival (A and B), Progression-free survival (C and D), disease control rate (E and F), Adverse events of grade 3 or higher (G and H), KRAS mutant (I and J), KRAS wild-type (K and L).

Abbreviations: OS, overall survival; PFS, progression-free survival; DCR, disease control rate; ≥3AEs, adverse events of grade 3 or higher.

A

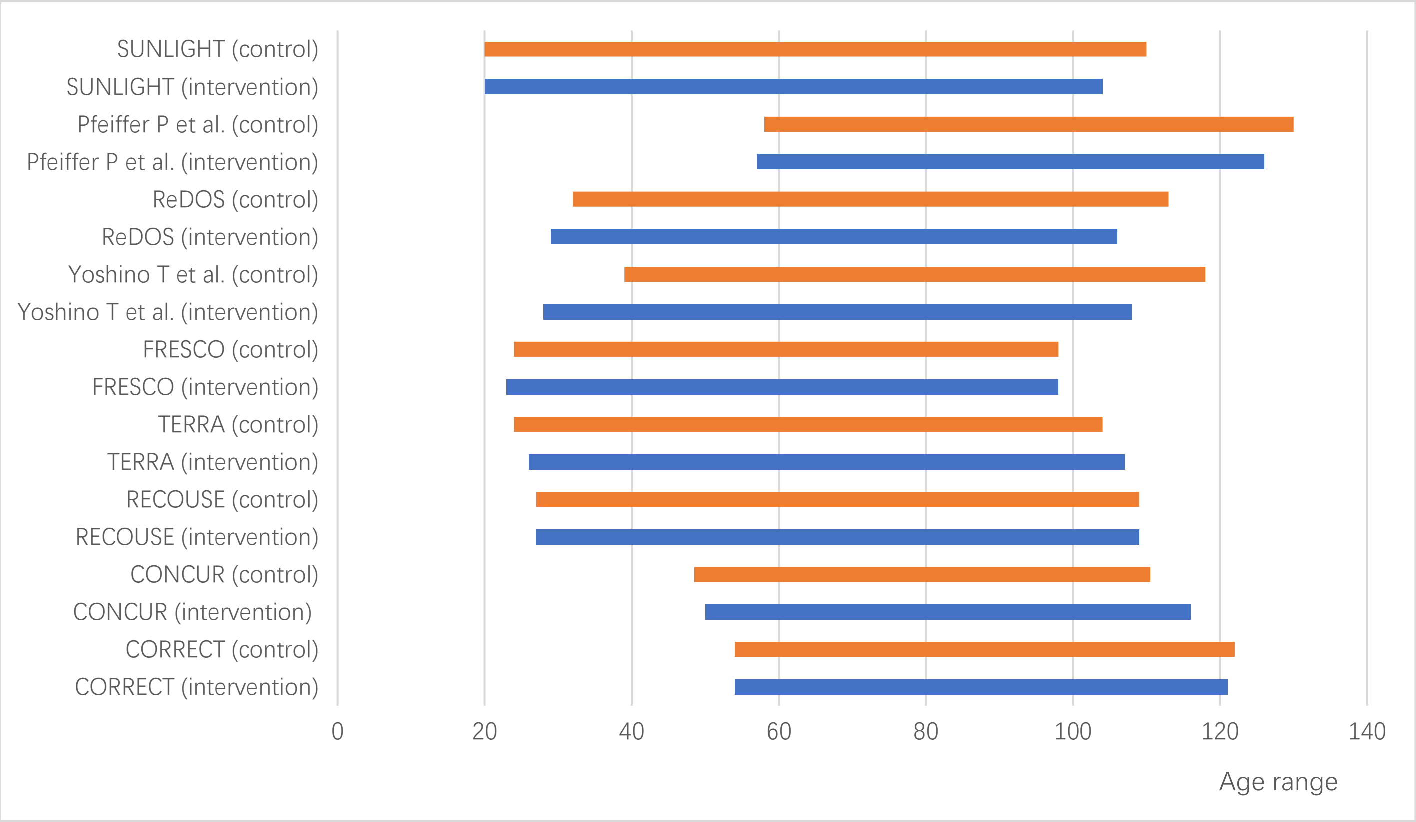

B

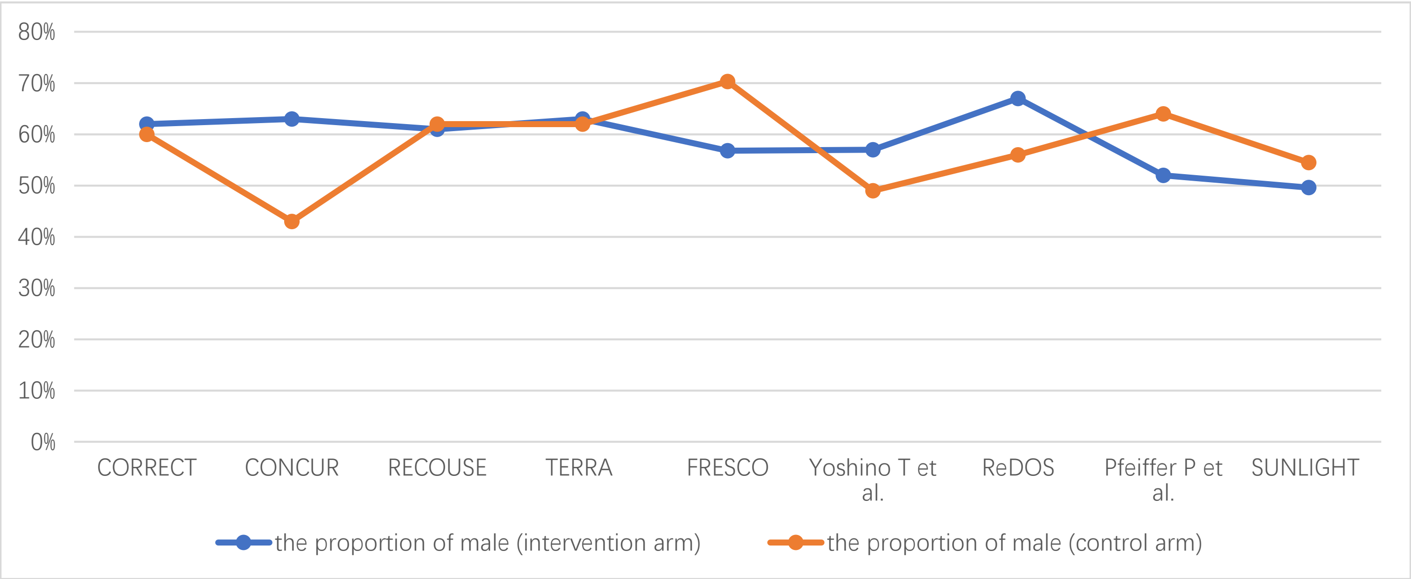

C

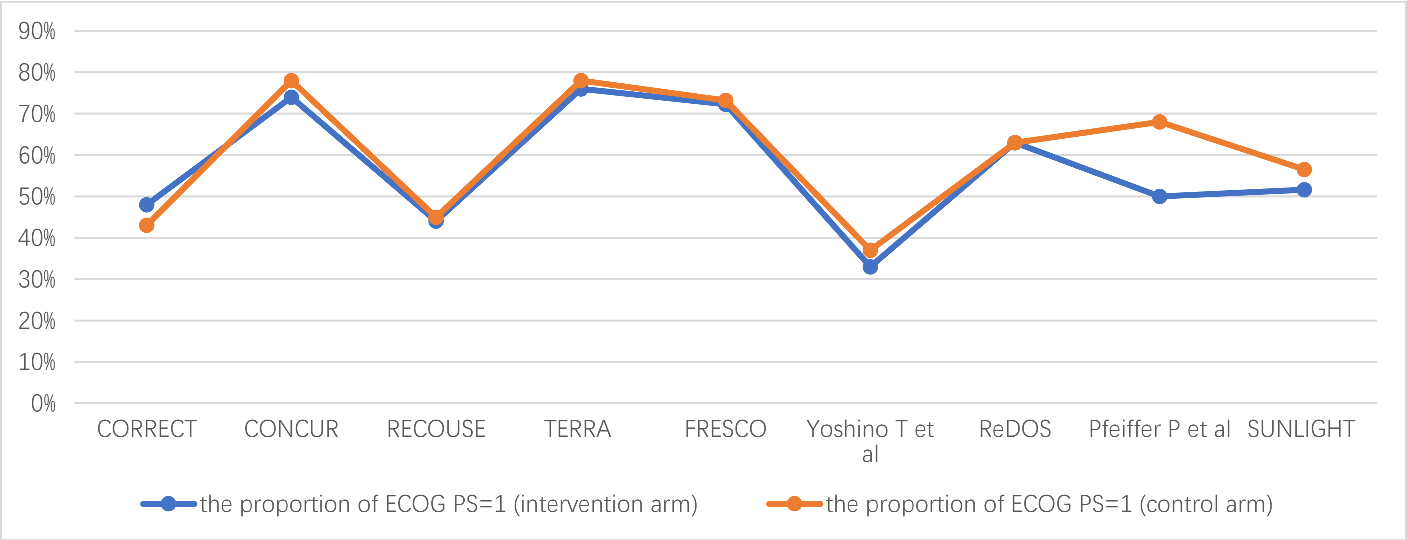

Supplementary Figure 7

Assessment of transitivity. (A) Median age and range of patients in intervention and control groups. (B) The proportion of male patients in intervention group and control group. (C)The proportion of ECOG PS=1 patients in intervention group and control group.

Abbreviations: ECOG PS, Eastern Cooperative Oncology Group Performance Status.
